# Genomic and Morphological Insights Into the Adaptive Evolution of Honeybees in Rocky Desertification Habitats

**DOI:** 10.1002/ece3.74169

**Published:** 2026-08-07

**Authors:** Yinglong Yu, Xiangyou Tang, Daoyin Chen, Ying Li, Dan Yao, Man Liu, Jinshan Xu, Xiaoping Wei

**Affiliations:** ^1^ Guizhou Institute of Animal Husbandry and Veterinary Science, Guizhou Academy of Agricultural Sciences Guiyang China; ^2^ College of Life Sciences, Chongqing Normal University Chongqing China; ^3^ Jinhua Academy of Agricultural Sciences Jinhua China; ^4^ Guizhou Institute of Integrated Agriculture Development, Guizhou Academy of Agricultural Sciences Guiyang China; ^5^ Guizhou Institute of Biology, Guizhou Academy of Sciences Guiyang China; ^6^ State Key Laboratory of Resource Insects, Southwest University Chongqing China

**Keywords:** honeybee, morphology, rocky desertification, selective sweep, whole‐genome resequencing

## Abstract

Unlike traditional desertification, rocky desertification is a distinct form of land degradation characterized by low vegetation coverage and a high proportion of rock exposure. Honeybees are crucial pollinators and ecological restorers and have coevolved closely with plants as their primary habitat and food resource to naturally inhabit rocky desertification habitats. In this study, classical morphological analyses showed that under mild desertification conditions, honeybee body size increases, while in severely degraded habitats, their morphology resembles that of normal individuals. Whole‐genome resequencing revealed clear selection effects on 19 genes, including *GBE*, *CerS, Schlank*, *ADO*, *Bicaudal,* and *Tim29*, which are potentially associated with glycogen and fatty acid metabolism for starvation resistance, substance transportation, and resistance. In addition, a genome‐wide association study (GWAS) identified 421 single nucleotide polymorphisms (SNPs) associated with 30 morphological traits in *Apis cerana* . These findings expand current understanding of the genetic response of honeybees in diverse environmental conditions and provide insight into how rocky desertification habitats shape insect morphology. This work offers a valuable reference for the molecular and morphological breeding of stress‐resistant honeybee strains and provides a novel approach to restoring plant diversity in desertification habitats from a pollinator perspective.

## Graphical abstract

Rocky desertification exerts significant selective pressures on honeybee morphology. Honeybees undergo adaptive evolution in 19 genes such as glycogen biosynthesis. Genes like GBE promote organismal survival under desertification.

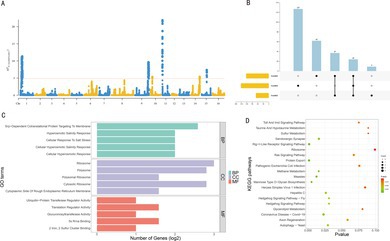

## 1 Introduction

Rocky desertification is a distinct form of desertification that predominantly occurs in humid carbonate areas (Zhu and Cui 1996). It is characterized by ecosystem degradation manifested through vegetation loss, soil and water loss, land quality decline, extensive rock exposure, and the emergence of desert‐like landscapes (Wang 2002; Xiong et al. 2002). These processes are typically driven by external factors such as agricultural expansion, deforestation, and geological disturbances in these ecologically fragile carbonate rock areas (Song et al. 2014).

As organisms are naturally subjected to selection pressures imposed by their local environments (Kawecki and Ebert 2004; Hancock et al. 2011), the species composition, individual density, and species diversity index of animals are generally reduced in rocky desertification habitats (Wang et al. 2011; Dai et al. 2019). Specifically, studies on insects have shown a significant negative correlation between the degree of rocky desertification and the structural characteristics of leafhopper communities, including decreased Shannon‐Wiener diversity and Margalef richness indices (Zhao et al. 2015; Wang et al. 2020).

The honeybee's ancestor (*Melittosphex burmensis*) dates back nearly 100 million years ago, and today honeybees are found on all the major continents except Antarctica (Garnery et al. 1992; Arias and Sheppard 1996; Han et al. 2012). Over its long evolutionary history and broad geographic distribution, honeybees have gained important ecological and economic value (Potts et al. 2016; Timberlake et al. 2026). They have also evolved distinct biological characteristics in diverse environments, including cold tolerance, propolis preference, and resistance to *Varroa* mites (Martin and Medina 2004; Fazier et al. 2010; Strauss et al. 2013). They are highly dependent on plants for both habitat and food, and their evolutionary history is deeply intertwined with that of plants. The development of specialized morphological characteristics, such as leg modifications and body hairs, that facilitate pollination as well as efficient collection of pollen and nectar are clear examples of this coevolution (Meixner et al. 2007). While it has been established that rocky desertification habitats directly influence plant communities by altering species composition, leaf structure, and physiological traits (Du et al. 2008; Rong et al. 2008; Xia et al. 2020), it remains largely unclear whether and to what extent rocky desertification affects plant‐dependent insects such as honeybees.

Therefore, this study explored whether honeybees in rocky desertification habitats are genetically distinct, to identify the genetic and morphological changes that may underlie their adaptation to these conditions. Results will not only enrich current understanding of honeybee adaptive evolution across diverse environments but also broaden insights into how rocky desertification shapes pollinator populations.

## 2 Materials and Methods

### 2.1 Sample Collection

**FIGURE 1:**
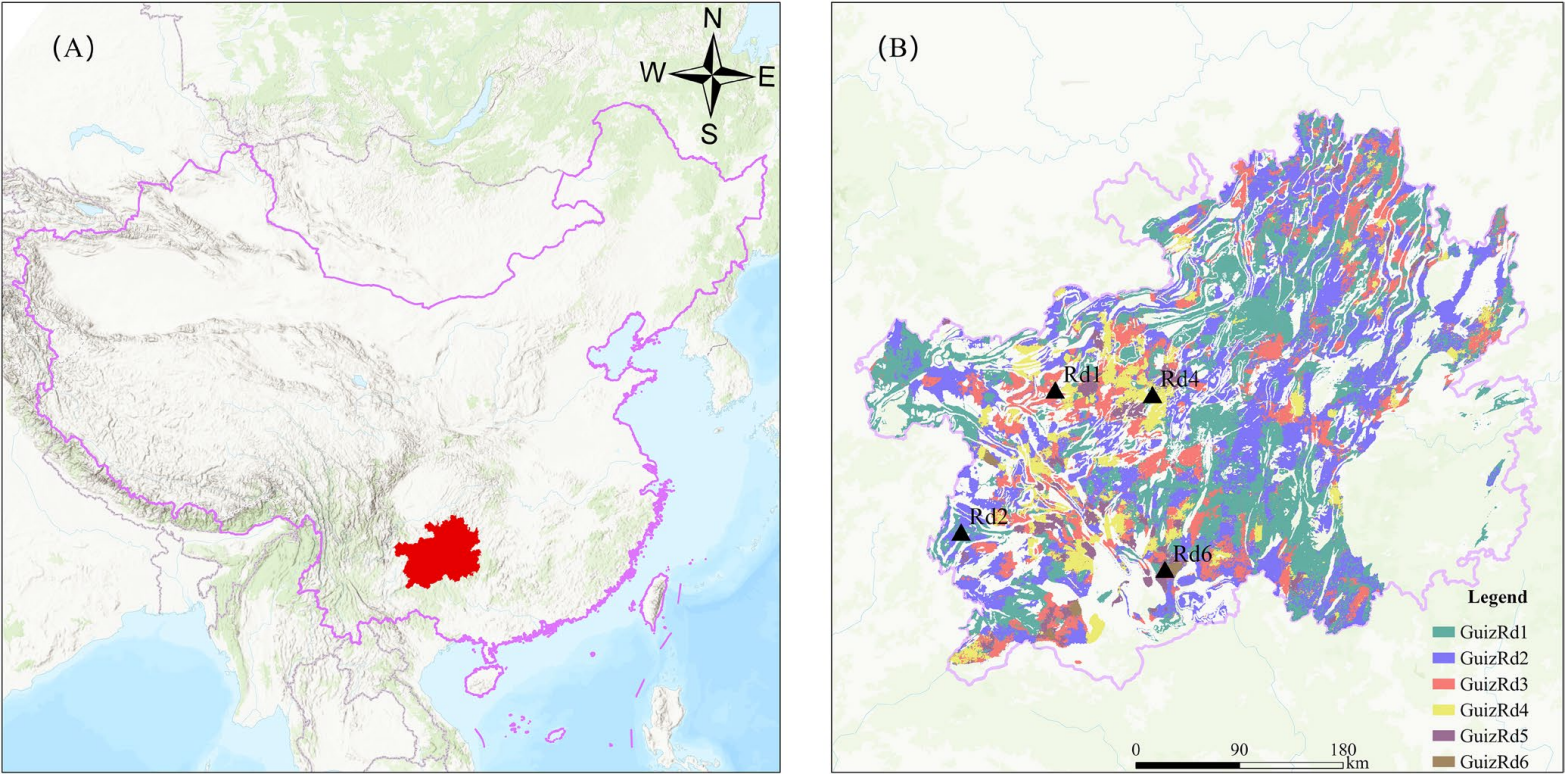
Geographic distribution of sampled honeybee colonies from rocky desertification habitats in China. (A) Geographical positions of the sampling sites within China. (B) Specific locations of the sampling sites and the distribution of rocky desertification habitats.

Rocky desertification habitats were sampled from Guizhou (China), a representative region where rocky desertification is widespread, and were classified into six categories based on criteria established primarily on climatic and ecological factors such as vegetation cover, soil cover, and soil thickness (Xiong et al. 2002). Degree one represents non‐rocky desertification (D1, control), degree two indicates potential rocky desertification (D2), degree three corresponds to slight rocky desertification (D3), degree four to moderate rocky desertification (D4), degree five to extreme rocky desertification (D5), and degree six to severe rocky desertification (D6). Given the difficulty of sample collection, samples could not be obtained from all degrees, and samples from D1, D2, D4, and D6 were selected as representatives, where D2–6 represent degrees of rocky desertification. The number of colonies sampled from each degree was 27, 31, 25, and 27, respectively, totaling 110 colonies, and hereafter referred to as GuizRd1, GuizRd2, GuizRd4, and GuizRd6, respectively (Figure 1). The distances between the four sample sites range from 84.3 to 204.0 km.

All honeybees were collected from traditionally farmed outdoor hives without movable frames, naturally populated by wild bees during the swarming season. Local farmers do not intervene in colony management, harvesting honey only once or twice a year, with no buying or selling of colonies. Consequently, these colonies are free from external disturbances such as artificial selection and can be regarded as semi‐wild. One worker bee was sacrificed from each colony for genomic resequencing and morphological experiments simultaneously. The genetic background of workers in a colony is derived from one queen and several drones, and honeybee workers are either maternal full sisters or half‐sisters. Therefore, in honeybee research, it is preferable to collect samples from a larger number of colonies rather than repeatedly sampling from a single colony to reduce the risk of pseudo replication (Chen et al. 2018; Ji et al. 2020; Shi et al. 2020).

Additionally, the corresponding environmental factors were retrieved for four distinct rocky desertification habitats from the National Earth System Science Data Center of China as a 40‐year annual series (1982–2021). A total of 17 environmental factors were included: net shortwave radiation, longwave radiation, near‐surface air temperature, total rainfall, total evapotranspiration, potential evapotranspiration, canopy interception evaporation, vegetation transpiration, specific humidity, total precipitation, soil heat flux, land surface temperature, soil temperature, groundwater runoff, soil water content, total soil moisture, and bare soil evaporation.

### 2.2 DNA Extraction, Genome Sequencing, Read Mapping, SNP Calling, and Annotation

Genomic DNA was extracted from the thoracic tissue of all sampled worker honeybees, and sequencing libraries were prepared using the NEB Next Ultra DNA Library Prep Kit (New England Biolabs, USA) following the manufacturer's instructions. Genomic DNA was sheared to an average fragment size of 350 bp, end‐polished, A‐tailed, and ligated to full‐length adapters before PCR amplification. Notably, libraries were assessed for size distribution in the Agilent 5300 and quantified by q‐PCR.

Whole‐genome sequencing was conducted on the Illumina Novaseq PE150 platform. Low‐quality paired reads were removed based on the following criteria: (i) reads containing adapter sequences; (ii) reads with ≥ 10% unidentified nucleotides (N); and (iii) reads with > 50% bases having a Phred quality score < 5 (Zhang et al. 2021). High‐quality reads were then aligned to the reference genome (GCA_020141525.2) in BWA v0.7.5 with the parameters “‐t 4 ‐k 32 ‐M” (Li and Durbin 2009). Duplicate reads (rmdup) were removed with SAMTOOLS v1.4 (Li et al. 2009), and SNP calling was conducted in SAMTOOLS v1.4 following a Bayesian approach, with the command Dp5‐miss 0.1‐maf 0.05 removing low‐quality SNPs. Finally, SNP annotation was performed relative to the reference genome (GCA_020141525.2) using ANNOVAR v20130520 (Wang et al. 2010). To provide a more comprehensive view of the genetic differentiation of *Apis cerana* , partial analyses incorporated whole‐genome data from an additional 96 honeybee colonies in China (Chen et al. 2018; Shi et al. 2020).

### 2.3 Population Structure, Genetic Diversity, and Differentiation

Population genetic structure was assessed in Admixture v1.3.0 with the number of assumed genetic clusters (K) ranging from 2 to 9 (Alexander et al. 2009). Principal component analysis (PCA) was conducted with the top three principal components using GCTA v1.94.1 to further evaluate genetic structure (Yang et al. 2011). Genetic diversity within populations was quantified by calculating the effective number of alleles (Ne), expected heterozygosity (He), observed heterozygosity (Ho), polymorphism information content (PIC), and nucleotide diversity (PI) in PLINK v1.9 (Purcell et al. 2007). Pairwise Fixation Index (*F*_ST_) values were calculated using the SNPRelate package in R v4.5.2 (Zheng et al. 2012).

### 2.4 Selective Sweep and Enrichment Analysis

To identify genomic regions potentially influenced by rocky desertification habitats, *F*_ST_ values were calculated in VCFtools v0.1.17 in 100‐kb sliding windows with a 10‐kb step size (Danecek et al. 2011). The *F*_ST_ parameter is the inbreeding coefficient of subpopulations and was used to estimate pairwise genomic differentiation among populations at candidate loci. The resulting *F*_ST_ results were standardized and transformed into *z*‐scores using the same sliding window parameters. Genomic regions within the top 1% of *z*‐scores were considered candidates for selection. Gene ontology (GO) annotation and Kyoto Encyclopedia of Genes and Genomes (KEGG) pathway datasets for the reference genomes were constructed in Eggnog‐mapper, and the GO enrichment and KEGG pathway analyses were then performed for the selected genes. The resulting *p* values were corrected for multiple comparisons using the false discovery rate (FDR) method.

### 2.5 Measurement and Statistical Analysis of Morphological Characteristics

In total, 33 morphological traits were measured to evaluate adaptive evolution as described by Zhu et al. (2017). Most traits were adopted from standardized western honeybee ( *Apis mellifera* ) measurements as proposed by Ruttner et al. (1978) and Ruttner (1988). However, measurements of pigmentation, cubital index, and the length of tergite three were modified to account for morphological differences between *A. cerana* and *A. mellifera* (Yang 2001).

Morphological data were statistically analyzed in R v4.5.2. Analysis of variance (one‐way ANOVA) was performed when Shapiro–Wilk tests of the residuals (normality) and Levene's test with median centering (homogeneity of variance) assumptions were met. Otherwise, a Kruskal–Wallis test was used. To control for false discovery rate across the 33 traits, raw *p* values were adjusted with the Benjamini–Hochberg procedure. Effect sizes were reported as *η* ^2^ (ANOVA) or *η* ^2^_*H* (Kruskal–Wallis). For traits significant after adjustment, post hoc pairwise comparisons used Tukey's HSD (parametric traits) or Dunn's test with Benjamini–Hochberg adjustment (non‐parametric traits). Non‐measurable values (proboscis length in 46 samples and isolated abdominal/venation characters in four damaged samples) were treated as missing rather than zero. Subsequently, multivariate analyses were performed on a dataset of 106 samples complete across the 32 measured traits (proboscis length was ultimately excluded owing to its high missing rate).

PCA and linear discriminant analysis (LDA) were conducted on *z*‐standardized morphological data to visualize differences among groups. Classification accuracy of the LDA was estimated by leave‐one‐out cross‐validation (LOO‐CV) and compared with the 25% four‐group chance level. Similarly, the assumption‐checked ANOVA/Kruskal–Wallis and Benjamini–Hochberg framework tested for among‐group differences for each environmental factor. The morphological matrix was compressed to its first two principal components (PC) (the 15 among‐group‐differing factors) to avoid overparameterization at four groups, whereafter a redundancy analysis (RDA) was performed, constrained by the environmental gradient. The constrained fraction was then tested by 999 permutations with the pseudo replication caveat noted. The geographic structuring of each morphological trait was quantified by a linear mixed model (LMM), and the among‐group variance proportion (intraclass correlation, ICC) was reported. Spearman correlations performed between the 13 differing morphological traits and 15 differing environmental factors were displayed as a heatmap.

### 2.6 Genome‐Wide Association Study

A genome‐wide association study (GWAS) was conducted using a multi‐locus random‐SNP‐effect mixed linear model (mrMLM) analysis implemented in the mrMLM package in R v4.5.2 (Zhou and Stephens 2014), while evolutionary population structure and PCA were controlled as fixed and random effects, respectively, to improve test efficacy. The effective number (*n*) of independent SNPs was calculated in GEC v0.2, and significance (*p* = 0.05/*n*, Bonferroni correction) was set to control the genome‐wide type I error rate. Ultimately, the significance *p* value was determined to be 5.47E‐8, which corresponds to −log_10_(*p*) = 7.26. Meanwhile, to ensure the reliability of significant association signals in the GWAS, only SNPs that appeared as significant in at least two models were considered credible loci. Considering linkage disequilibrium blocks (LD, the nonrandom association of alleles at different loci) surrounding significantly associated SNPs, the boundaries of candidate regions were defined in PLINK v1.9 (Slatkin 2008). Finally, Manhattan and quantile‐quantile plots were generated in the qqman package.

## 3 Results

The whole‐genome resequencing of 110 honeybee workers generated 608.30 Gb of clean data, with quality scores of Q20 ≥ 94.69% and Q30 ≥ 87.64%. The average mapping rate with the reference genome was 85.08% ± 3.13%, with an average sequencing depth (excluding the gap region) of 17.41X ± 3.16X and an average coverage of 97.69% ± 1.99% (at least one base covered). After quality control, a total of 1,074,039 SNPs were identified, with 88,483 located in exonic regions and 9906 classified as nonsynonymous.

### 3.1 Genetic Diversity and Divergence of Honeybees

**FIGURE 2:**
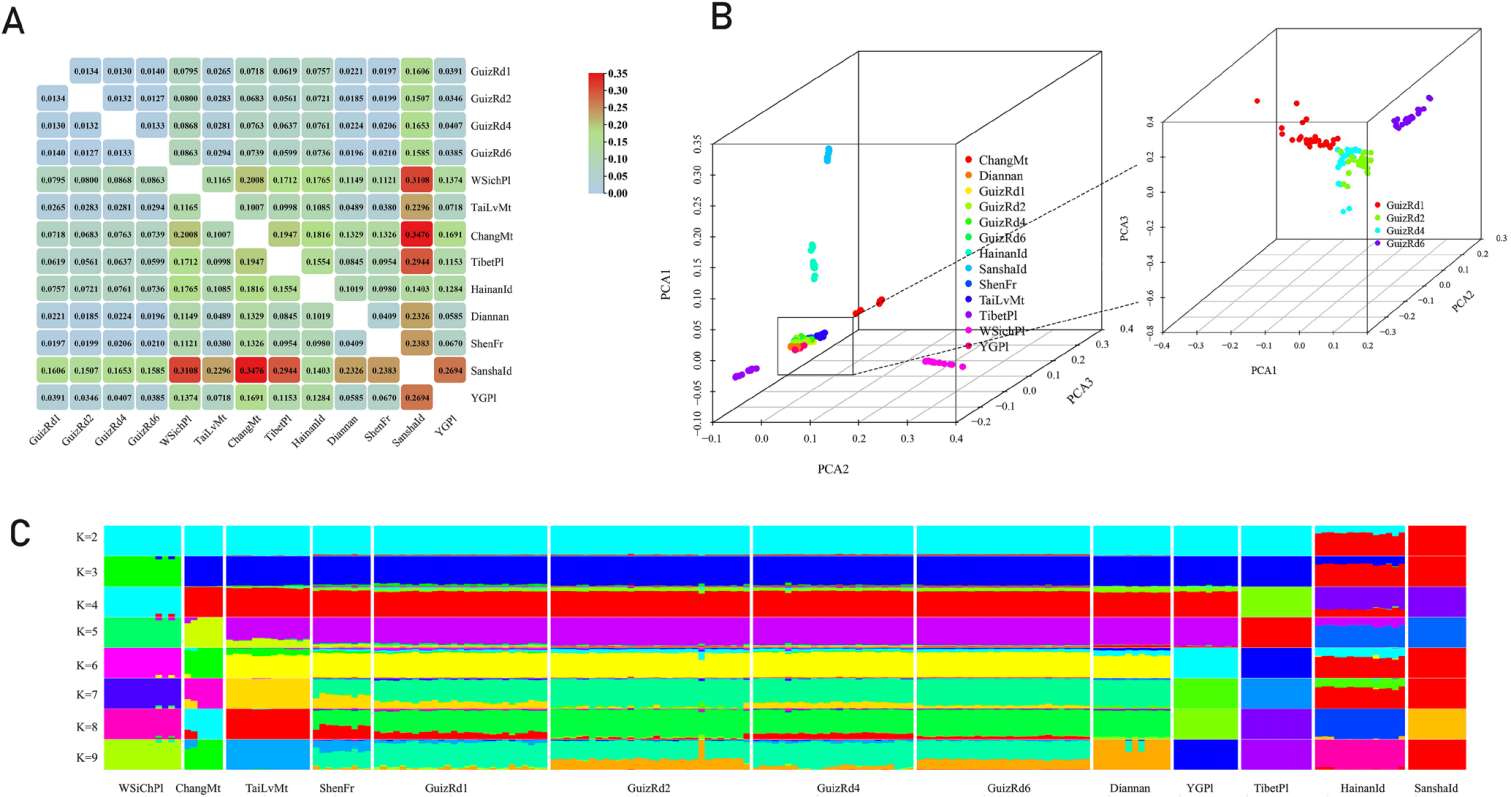
Genetic analysis of eastern honeybees across various rocky desertification habitats in China based on whole‐genome and morphological data. (A) Pairwise *F*_ST_ values among honeybee populations. (B) Principal component analysis (PCA). (C) Population structure analysis based on high‐quality SNPs.

Honeybee genetic diversity in rocky desertification habitats was evaluated through five genetic diversity indicators: effective number of alleles (Ne), polymorphism information content (PIC), probability of identity (PI), observed heterozygosity (Ho), and expected heterozygosity (He) (Table 1). No significant differences in genetic diversity were observed among the four populations across varying degrees of desertification. This was corroborated by the pairwise genetic differentiation index, showing minimal genetic differentiation among populations (average *F*_ST_ = 0.0133) (Figure 2A).

**TABLE 1:** Genetic diversity of honeybees sampled from different colonies in Guizhou rocky desertification habitats.

Populations	Ne	Ho	He	PIC	PI
GuizRd1	1.397	0.257	0.253	0.168	0.746
GuizRd2	1.394	0.251	0.251	0.167	0.748
GuizRd4	1.398	0.258	0.254	0.168	0.746
GuizRd6	1.401	0.265	0.255	0.169	0.745
ChangMt	1.552	0.316	0.329	0.203	0.671
Diannan	1.429	0.230	0.270	0.177	0.729
HainanId	1.498	0.254	0.300	0.189	0.699
ShenFr	1.451	0.244	0.282	0.184	0.717
TaiLvMt	1.448	0.267	0.279	0.180	0.721
TibetPl	1.518	0.245	0.311	0.194	0.688
WsichPl	1.431	0.264	0.267	0.173	0.732
YGPl	1.491	0.254	0.299	0.191	0.699

*Note:* All regions are located in China.

Abbreviations: ChangMt, the Changbai Mountains; HainanId, Hainan Island; ShenFr, Shennongjia Forestry District; TaiLvMt, Taihang and Lvliang Mountains; TibetPl, the Tibetan Plateau; WSichPl, the West Sichuan Plateau; YGPl, the Yunnan‐Guizhou Plateau.

The ancestral genetic composition of 206 Chinese honeybee samples was analyzed using an admixture model, with the number of ancestral populations (*K*) ranging from 2 to 9 (Figure 2C). At *K* = 9, the nine previously reported ecotypes were clearly delineated, including populations from Aba, Changbai Mountain, Northern China, Central China, South Yunnan, the Yunnan‐Gui Plateau, Tibet, Hainan, and Sansha. The PCA is consistent with these results (Figure 2B), further corroborating observed genetic patterns. Notably, the PCA restricted to samples from rocky desertification regions revealed genetic differentiation among the four regions, where samples from D2 and D4 clustered together, and those from D1 and D6 formed distinct groups. Pairwise genetic differentiation indices (*F*_ST_) among these populations ranged from 0.0127 to 0.0134, with a mean of 0.0132 (Figure 2A).

**FIGURE 3:**
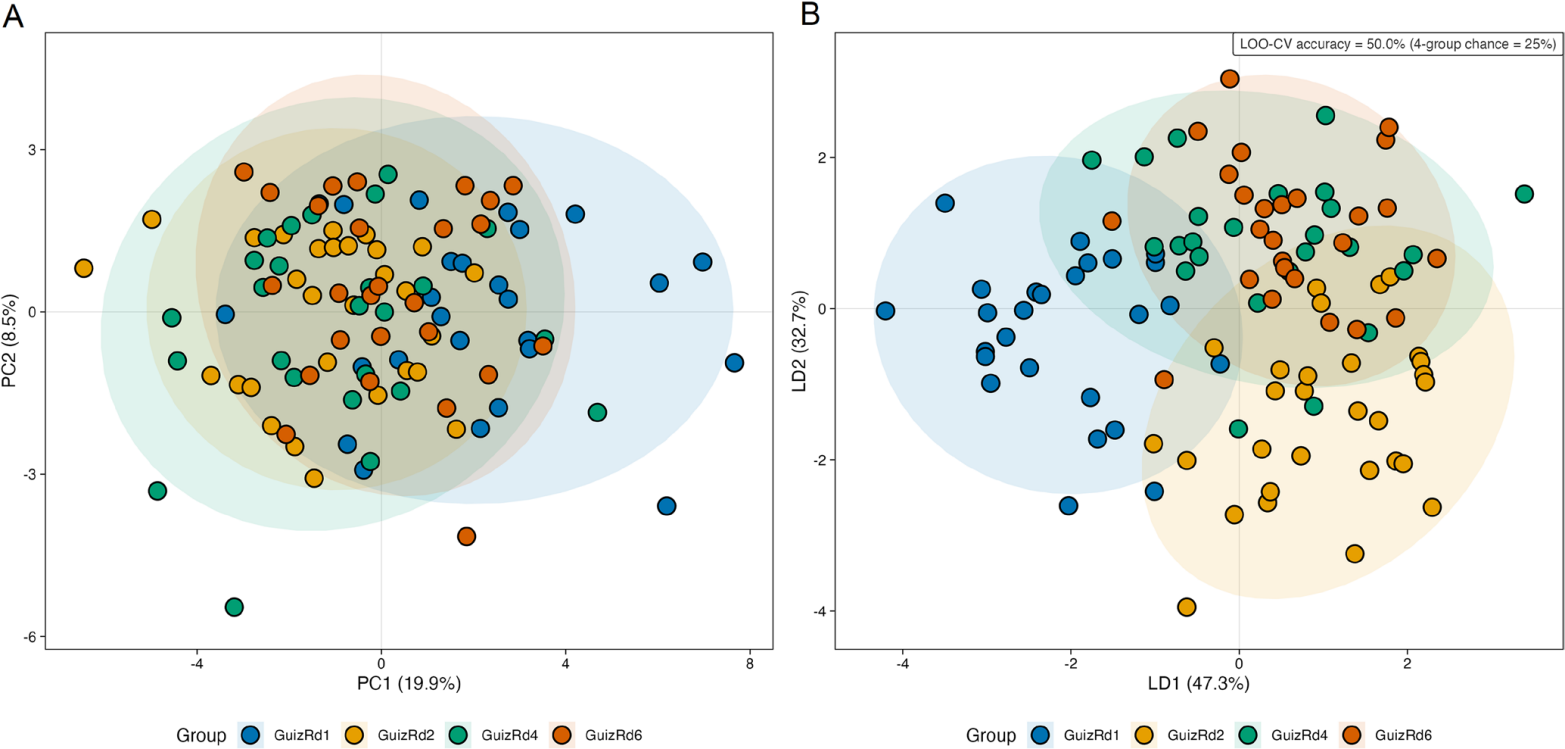
Genetic analysis of eastern honeybees across various rocky desertification habitats in China based on morphological data. (A) Principal component analysis. (B) Scatter plot of linear discriminant analysis.

After Benjamini–Hochberg adjustment, 14 of the 33 morphological traits differed significantly among the four groups (26 traits met parametric assumptions and were tested by ANOVA, while seven were tested by Kruskal–Wallis). These included proboscis length, forewing length, forewing width, vein angle E9, femur length, tergite 3 longitudinal length, tergite 4 longitudinal length, tergite 5 longitudinal length, tergite 6 longitudinal length, length of cover hair on tergite 6, sternite 4 longitudinal length, transverse length of wax plate on sternite 4, sternite 7 longitudinal length, sternite 7 transverse length (Table 2, Table S1). The PCA showed groups overlapping substantially, where the first two principal components accounted for 19.9% and 8.5% of the total morphological variance (Figure 3A), while the LDA separated the groups with a leave‐one‐out cross‐validation classification accuracy of 50.0% (≥ 25% four‐group chance) (Figure 3B). Honeybees from GuizRd1 exhibited the smallest values for 12 of the 14 length‐related morphological traits but had the largest values for one vein angle. In contrast, the GuizRd2, GuizRd4, and GuizRd6 colonies had relatively larger body sizes, with GuizRd2 and GuizRd4 scoring second in length for 12 of the 14 morphological traits (Table 2).

**TABLE 2:** Morphological analysis of variance (ANOVA) of honeybees from various rocky desertification habitats in China. Rows in bold font indicate significant morphological differences. The letters after the numbers: the same indicate no difference, while different indicate significant differences.

Group_Name	GuizRd1	GuizRd2	GuizRd4	GuizRd6
**Proboscis length**	**5.1664** **± 0.1087bc**	**5.2161 ± 0.1157c**	**5.0304 ± 0.1176a**	**5.0956 ± 0.0911ab**
**Forewing length**	**8.4608 ± 0.1846a**	**8.6812 ± 0.1644b**	**8.5662 ± 0.1973ab**	**8.4821 ± 0.1339a**
**Forewing width**	**2.9400 ± 0.0824a**	**3.0303 ± 0.0605c**	**3.0029 ± 0.0650bc**	**2.9631 ± 0.0616ab**
Vein angle A4	30.3 ± 1.6a	30.3 ± 1.8a	31.2 ± 1.2a	31.3 ± 2.5a
Vein angle B4	108.9 ± 5.1a	109.7 ± 4.0a	108.0 ± 4.2a	108.0 ± 4.1a
Vein angle D7	97.0 ± 2.7	96.2 ± 2.8	94.6 ± 3.4	95.4 ± 2.7
**Vein angle E9**	**78.0 ± 1.7c**	**74.9 ± 2.8ab**	**76.4 ± 2.7bc**	**74.5 ± 2.6a**
Vein angle J10	85.7 ± 3.1a	85.8 ± 3.0a	84.6 ± 2.9a	86.4 ± 3.5a
Vein angle L13	31.4 ± 3.7a	31.2 ± 3.5a	30.5 ± 4.2a	30.7 ± 3.6a
Vein angle J16	83.7 ± 2.3a	83.7 ± 3.7a	82.0 ± 3.4a	84.3 ± 3.9a
Vein angle G18	103.5 ± 2.8a	103.5 ± 3.1a	102.8 ± 3.1a	103.5 ± 3.2a
Vein angle K19	46.8 ± 2.9a	46.5 ± 3.2a	46.1 ± 2.6a	46.9 ± 2.6a
Vein angle N23	20.1 ± 1.4a	20.8 ± 1.4a	20.3 ± 1.2a	20.1 ± 0.8a
Vein angle O26	13.2 ± 1.7a	13.0 ± 1.2a	13.6 ± 1.6a	13.0 ± 1.4a
**Femur length**	**2.4597 ± 0.0745a**	**2.5387 ± 0.0638c**	**2.5166 ± 0.0721bc**	**2.4867 ± 0.0823ab**
Tibia length	2.9402 ± 0.1034a	2.9893 ± 0.0744a	2.9883 ± 0.1150a	2.9426 ± 0.0777a
Metatarsus length	1.9376 ± 0.0733a	1.9721 ± 0.0550a	1.9643 ± 0.0783a	1.9265 ± 0.0621a
Metatarsus width	1.0221 ± 0.0439a	1.0514 ± 0.0424a	1.0332 ± 0.0626a	1.0320 ± 0.0545a
**Tergite 3 longitudinal length**	**1.9699 ± 0.0845a**	**2.0682 ± 0.0594b**	**2.0263 ± 0.0888b**	**2.0296 ± 0.0826b**
**Tergite 4 longitudinal length**	**1.7739 ± 0.0456a**	**1.8185 ± 0.0379b**	**1.8266 ± 0.0548b**	**1.8030 ± 0.0466ab**
**Tergite 5 longitudinal length**	**1.7356 ± 0.0602a**	**1.7727 ± 0.0638ab**	**1.7856 ± 0.0464b**	**1.7588 ± 0.0485ab**
The width of the stripe of tomentum on Tergite 5	1.0111 ± 0.1232a	1.0551 ± 0.1099a	1.0100 ± 0.1037a	0.9744 ± 0.1359a
**Tergite 6 longitudinal length**	**1.7067 ± 0.1071a**	**1.8161 ± 0.0473b**	**1.7470 ± 0.1345a**	**1.7714 ± 0.0787ab**
**Length of cover hair on Tergite 6**	**0.4306 ± 0.0909a**	**0.5131 ± 0.0858b**	**0.4948 ± 0.0891b**	**0.4525 ± 0.0812ab**
**Sternite 4 longitudinal length**	**2.4747 ± 0.0756a**	**2.5262 ± 0.0727b**	**2.5491 ± 0.0641b**	**2.5246 ± 0.0555b**
Length of wax plate on Sternite 4	1.1379 ± 0.0562	1.1539 ± 0.0574	1.1802 ± 0.0552	1.1505 ± 0.0408
**Transverse length of wax plate on Sternite 4**	**2.0882 ± 0.0586a**	**2.1342 ± 0.0730b**	**2.1584 ± 0.0669b**	**2.1272 ± 0.0509ab**
Distance between wax plate on Sternite 4	0.2851 ± 0.0532a	0.3029 ± 0.0609a	0.3047 ± 0.0518a	0.2976 ± 0.0422a
**Sternite 7 longitudinal length**	**2.2168 ± 0.0670a**	**2.2672 ± 0.0695b**	**2.2786 ± 0.0560b**	**2.3099 ± 0.0760b**
**Sternite 7 transverse length**	**2.7303 ± 0.0807a**	**2.7528 ± 0.0787ab**	**2.7989 ± 0.0833b**	**2.7873 ± 0.0642b**
Cubital vein A	0.5369 ± 0.0412a	0.5550 ± 0.0364a	0.5552 ± 0.0501a	0.5483 ± 0.0412a
Cubital vein B	0.1485 ± 0.0233a	0.1445 ± 0.0274a	0.1405 ± 0.0219a	0.1497 ± 0.0235a
Cubital Index	3.7218 ± 0.7698a	3.9828 ± 0.8499a	4.0777 ± 0.9297a	3.7798 ± 0.8211a

**FIGURE 4:**
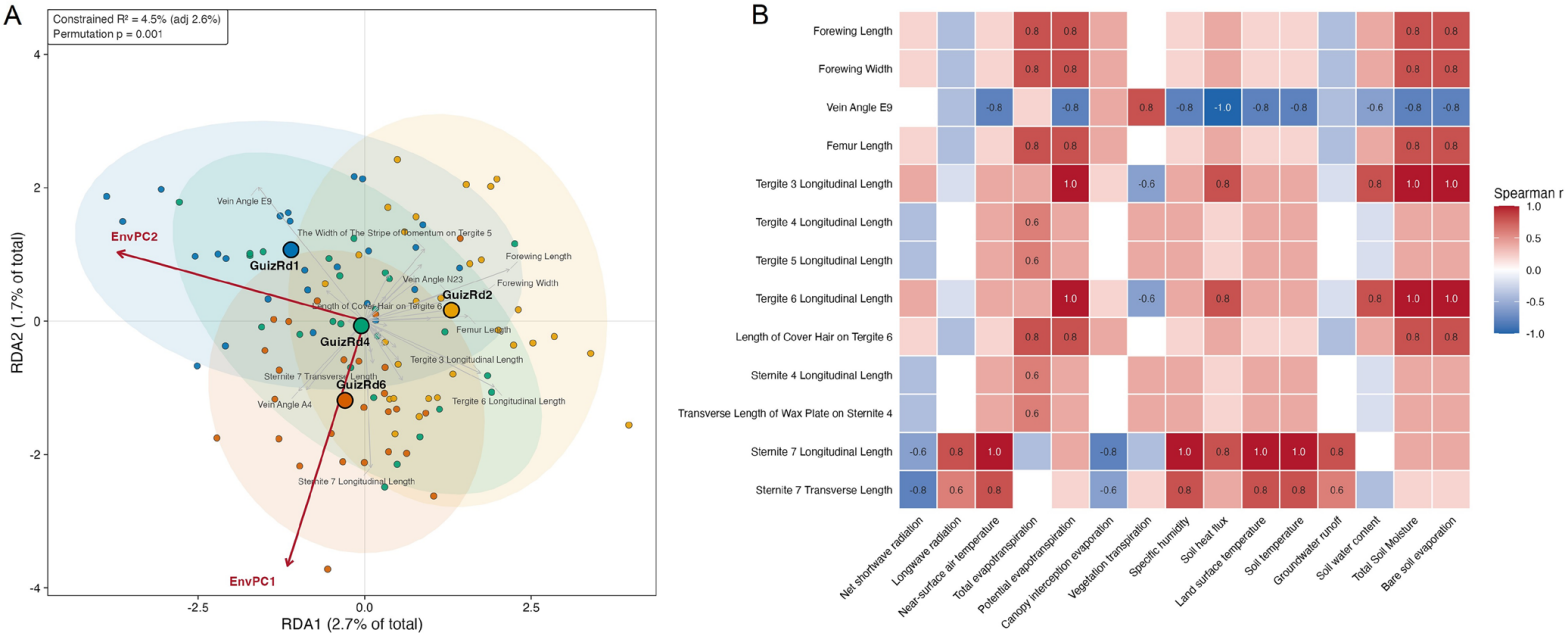
Potential effects of environmental factors on honeybee morphology in rocky desertification habitats. (A) Redundancy analysis of the relationship between environmental factors and morphological traits. (B) Spearman correlation heatmap of differential morphological traits and environmental factors.

Fifteen of the 17 environmental factors differed significantly among the four groups after Benjamini–Hochberg adjustment, with only total rainfall and total precipitation being insignificant. The environment‐constrained RDA explained a small fraction of morphological variation (constrained *R* ^2^ = 4.5%, adjusted 2.6%; permutation *p* = 0.001), consistent with a weak environmental signal given the four‐group resolution (Figure 4A). Similarly, the LMM attributed a modest median of 6% of the morphological variance to among‐group structure (Figure S1). Spearman correlations between differing traits and factors were moderate‐to‐strong in magnitude, but at the four‐group resolution, they were descriptive only and were not significance‐tested (Figure 4B). Thus, at the four‐group resolution, environmental gradients only explained a small fraction of morphological variation.

### 3.2 Gene Selection Under Rocky Desertification

To identify genomic regions potentially associated with the adaptation of Chinese honeybees to rocky desertification habitats, *F*_ST_ values were calculated across multiple population comparisons using VCFtools. Specifically, comparisons were made between D6 and D1, D4 and D1, and D2 and D1 populations. The resulting *F*_ST_ values were standardized into *z*‐scores, and genomic regions falling within the top 1% of *z*‐scores were designated as candidate selective regions.

**FIGURE 5:**
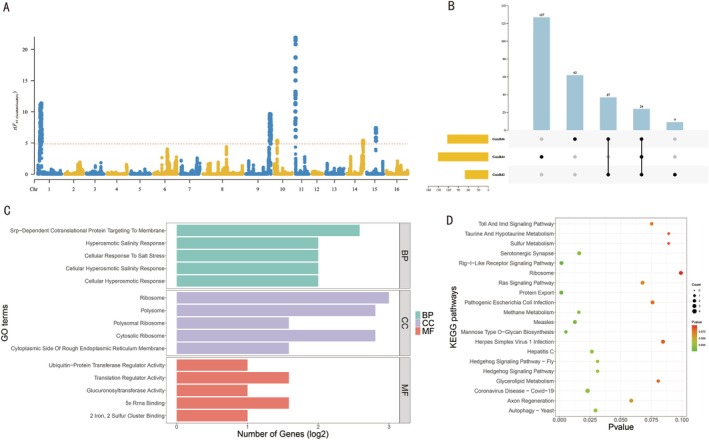
Natural selection in eastern honeybees from rocky desertification habitats in China. (A) Selective sweep analysis based on whole‐genome sequencing of GuizRd6. (B) Shared candidate genes identified across three levels of rocky desertification. (C) GO enrichment analysis of selected genes in GuizRd6. (D) KEGG enrichment analysis of selected genes in GuizRd6.

As a result, 123 candidate genes were identified in the D6 versus D1 comparison, 151 in the D4 versus D1 comparison, and 70 in the D2 versus D1 comparison (Figure 5B). Among these, 24 genes were shared across all 3 comparisons and were successfully annotated to 19 unique genes in NCBI, including *GBE* (1, 4‐alpha‐glucan‐branching enzyme, ID: LOC107992918), *CerS Schlank* (ceramide synthase schlank, ID: LOC107992919), *Bicaudal* (ID: LOC107993529), *Tim29* (mitochondrial import inner membrane translocase subunit, ID: LOC107992922), and *ADO* (2‐aminoethanethiol dioxygenase, ID: LOC107993536). Compared with other desertification habitats, honeybees from rocky desertification habitats exhibited significant differences in enriched pathways, including mannose‐type O‐glycan biosynthesis, starch and sucrose metabolism, sphingolipid metabolism, apoptosis (fly), and neuroactive ligand‐receptor interaction (Figure 5C,D).

### 3.3 Genome‐Wide Association Studies

The GWAS identified 421 SNPs significantly associated with 30 morphological markers, including proboscis length, forewing length and width, vein angles (A4, B4, D7, E9, J10, L13, J16, G18, K19, N23, and O26), tibia length, femur length, metatarsus length, metatarsus width, longitudinal lengths of tergite 3–6, width of the tomentum stripe on tergite 5, length of cover hairs on tergite 6, longitudinal length of sternite 4, length and transverse length of wax plate on sternite 4, distance between wax plates on sternite 4, and longitudinal length and transverse length of sternite 7 (Table S2). Among these, 350 SNPs were effectively annotated to 323 genes known to play critical roles in insect morphological development, including pupal cuticle protein (*PCP*), elongation of very long‐chain fatty acids protein (*elovl*), and transforming growth factor beta regulator (*TGF‐β*).

## 4 Discussion

This integrated molecular and morphological study revealed that honeybees inhabiting rocky desertification habitats have undergone genetic changes associated with transportation, glycogen biosynthesis, as well as body fat regulation and resistance, which may enhance their ability to survive in environments with limited nectar sources. Studies examining insect‐environment interactions shed light on the potential genetic adaptations of honeybees to rocky desertification habitats and highlight their inherent diverse distribution and adaptability as a ubiquitous pollinator. The natural establishment and expansion of honeybee support vegetation regeneration, thereby offering a promising new avenue for the ecological remediation of rocky desertification habitats.

### 4.1 Genetic Differentiation

The average *F*_ST_ among *A. cerana* subspecies is approximately 0.162 (0.099–0.228) (Chen et al. 2018), whereas that among *A. mellifera* subspecies is about 0.10 (0.05–0.15) (Wallberg et al. 2014). This study detected weak genetic differentiation (*F*_ST_ < 0.01) among the four honeybee groups, contrary to initial predictions. Notably, GuizRd1 showed a relatively higher *F*_ST_ compared with the other populations, suggesting that rocky desertification does not generate independent populations, but exerts an influence on honeybee population structure. Also, fewer candidate genes were identified in these populations compared with studies conducted on populations from high altitude, high sunlight, and low temperature environments (Chavez‐Galarza et al. 2013; Henriques et al. 2018; Montero‐Mendieta et al. 2019; Ji et al. 2020; Shi et al. 2020).

The absence of a unique rocky desertification habitat‐adapted population might be due to a lack of substantial physical barriers (Techer et al. 2017; Aglagane et al. 2022; Janashia et al. 2025), as rocky desertification habitats of different grades are interspersed, and large continuous areas of the same grade are rare. Given the migration ability of honeybees (Schneider and McNally 1994; Villa 2004; Jensen et al. 2005), it is likely that individuals adapted to a particular habitat remain there, while non‐adapted individuals readily migrate to adjacent areas. Moreover, the selective pressures exerted by rocky desertification habitats on honeybee populations appear weaker than the strong selective pressure observed in classic cases, such as the body color change of *Biston betularia* (Tian et al. 2024).

### 4.2 Genes in Selective Sweeping

It was originally hypothesized that rocky desertification habitats may influence honeybees in two main ways, and these candidate genes and pathways were partially consistent with these assumptions. First, the reduction of nectar plants could enhance traits related to foraging efficiency, such as increased flight ability (greater distance or efficiency) and heightened sensory abilities (improved olfaction and vision for locating nectar sources). Second, the scarcity of nectar plants implies limited food resources, which may drive adaptations in nutritional metabolism, stress resistance, and other physiological processes that improve survival under conditions of food shortage. Here, several candidate genes were associated with glycogen and fatty acid metabolism for starvation resistance, substance transportation, and resistance. For example, *GBE* (1, 4‐alpha‐glucan‐branching enzyme, ID: LOC107992918) is involved in glycogen metabolism and directly influences starch structure, where *GBE* mutations can cause fatal glycogen storage disorders (Huynh et al. 2019). Specifically, *GBE* cleaves 1,4‐α‐glucan to form α‐1,6‐glucosidic linkages, where this branching of glycogen chains increases solubility and, consequently, affects biological functions (Akman et al. 2006; Liu et al. 2017). Similarly, *CerS Schlank* (ceramide synthase Schlank, ID: LOC107992919) functions both as an enzyme and as a transcriptional regulator, playing a central role in lipid homeostasis (Sociale et al. 2018). It has been implicated in body fat regulation and growth control in *Drosophila* (Bauer et al. 2009; Bauer 2010; Kraut 2011), and fatty acids are thought to increase the levels of *CerS Schlank* as nuclear transcription factors, whereas starvation is believed to decrease *CerS Schlank* levels (Sociale et al. 2018). Thus, the selective sweep reflected by these two genes may be associated with the honeybees' response to starvation in rocky desertification habitats.

Also, *ADO* (2‐aminoethanethiol dioxygenase, ID: LOC107993536) is involved in taurine biosynthesis (Dominy et al. 2007), which affects mitochondrial bioenergetics and improves physical performance (Seidel et al. 2019). Specifically, taurine feeding has been shown to improve the growth, development, lifespan, immunity, and antioxidant capacity of honeybees under high temperature stress (Chen et al. 2023). Similarly, *Bicaudal* (ID: LOC107993529) is an important dynein adaptor responsible for transport (especially mRNA) along microtubules (MTs), and is closely associated with apoptosis in cells (Claussen and Suter 2005; Bullock 2007; Creagh et al. 2009). The mitochondrial import inner membrane translocase subunit, *Tim29* (ID: LOC107992922), is an inner membrane translocase of the human *TIM22* complex that facilitates the movement of multipass transmembrane proteins into the mitochondrial inner membrane (Rehling et al. 2003; Callegari et al. 2016). Meanwhile, *Tim29* interacts with the translocase of the outer mitochondrial membrane or TOM complex, influencing the transport of hydrophobic carrier substrates (Jackson et al. 2021). Notably, although these genes display pronounced selection signatures at the genomic level, their active contributions in honeybee adaptation await further experimental validation.

Contrary to initial hypotheses, as the degree of rocky desertification intensifies, honeybee body size increased, and finally decreased to normal at D6. The relationship between organismal body size and factors affecting it, such as temperature, is usually not simply linear; instead, there exists an optimal neutral zone, where any deviation can inhibit growth (Habeeb et al. 2023). Additionally, multiple environmental factors often act in concert (Keating‐Bitonti and Payne 2016; Ramos‐Rodríguez et al. 2020; Watson and Kerr 2025), including temperature, altitude, food availability, and habitat, and the cumulative interaction of these multiple factors can result in a distinct body size phenotype in a given environment (Ameline et al. 2026).

Although morphological changes correlated with the environment on the heatmap, further attention and supporting evidence are needed, given that only four sampling sites were included in this study. Based on the correlation heatmap, humidity and temperature had the largest effect on honeybee morphology, a finding supported by the literature (Coroian et al. 2014). Notably, humidity and temperature can influence honeybee mating to some degree, and may even affect genetic differentiation at the subspecies level (Polatto et al. 2014).

### 4.3 Genome‐Wide Association Studies

Genome‐wide association analysis identified 421 SNPs associated with 30 morphological markers, annotated to 323 genes, including pupal cuticle protein (*PCP*), elongation of very long‐chain fatty acids protein (*elovl*), and transforming growth factor beta regulator (*TGF‐β*). Specifically, *PCP* is a crucial protein in insect morphological development, regulating the sclerotization process and directly influencing body color by regulating pigment metabolism and complex formation (Hopkins et al. 2000; Yang et al. 2019; Kang et al. 2023; Jin et al. 2024). Similarly, *Elovl* contributes to epidermal barrier construction and lipid metabolism by synthesizing very long‐chain fatty acids (*Vlcefa*), which indirectly affect pigment stability or carrier function (Oboh et al. 2017; Kong et al. 2020), while *TGF‐β* is involved in the regulation of insect morphological development, including dorsoventral axis differentiation, appendage development during embryogenesis (Wang et al. 2024), and regulation of ecdysone and chitin metabolism during metamorphosis (Nonninger et al. 2025). Thus, these GWAS results provide a preliminary basis for future molecular‐level breeding of the honeybee. However, two limitations are acknowledged: first, a large number of SNPs were identified in this study, and second, many SNPs were annotated to genes not directly related to insect morphology. Therefore, future studies are required to validate the functional relevance of these SNPs.

## 5 Conclusions

Findings provide preliminary insights into the genetic influence of rocky desertified habitats with scarce food sources on their key pollinator. In the rocky desertification habitats, honeybees generally exhibit larger body sizes, although individuals in the most severe rocky desertification habitats display body sizes closer to normal. Preliminary analyses identified 19 key genes potentially associated with transportation, glycogen biosynthesis, body fat regulation, and stress resistance under selective sweep in rocky desertification populations. This work offers potentially valuable information for molecular and morphological breeding aimed at enhancing stress resistance in natural pollinators.

## Author Contributions

**Yinglong Yu:** conceptualization (equal), data curation (equal), investigation (equal), methodology (equal), supervision (equal), visualization (equal), writing – original draft (equal). **Xiangyou Tang:** data curation (equal), methodology (equal), visualization (equal). **Daoyin Chen:** data curation (equal), methodology (equal). **Ying Li:** investigation (equal). **Dan Yao:** investigation (equal), methodology (equal). **Man Liu:** methodology (equal). **Jinshan Xu:** methodology (equal), supervision (equal). **Xiaoping Wei:** conceptualization (equal), methodology (equal), supervision (equal).

## Funding

This study was supported by Guizhou Provincial Science and Technology Project (no. ZK[2024]079, no. [2024]097), China Agriculture Research System of MOF and MARA (no. CARS‐44‐SYZ 22), State Key Laboratory of Resource Insects (SKLRI‐ORP202413), Guizhou Academy of Agricultural Sciences (no. 202313), Guizhou Provincial Special Economic Animals Sci. & Tech. Innovation Leading Talent Workstation (KXJZ[2024]023), and the Guizhou Provincial Sci. & Tech. Innovation Leading Talent Project.

## Conflicts of Interest

The authors declare no conflicts of interest.

## Supporting information


**Table S1:** Information of morphological analysis of variance of honeybees in various rocky desertification habitats.
**Table S2:** Genome‐wide association study of morphological traits in honeybees from rocky desertification habitats in China.
**Figure S1:** Linear mixed model (LMM) estimation of proportional variance among‐groups and within‐groups based on honeybee morphology in rocky desertification habitats.

## Data Availability

Individual genotype data are available on the NCBI database (PRJNA1293808: SAMN50045067‐SAMN50045176).
