# Supplementary material for: Genomic and Morphological Insights Into the Adaptive Evolution of Honeybees in Rocky Desertification Habitats
Source: Ecol Evol. 2026 Aug 7;16(8):e74169. doi: 10.1002/ece3.74169 (PMC13451161; doi:10.1002/ece3.74169)
Supplement: Supplementary file 1 [file ECE3-16-e74169-s001.docx]

**TABLE S1** | Information of morphological analysis of variance of honeybees in various rocky desertification habitats.

| Trait | N | Normality Shapiro P | Homogeneity Levene P | Test | P raw | P BH | Effect | Effect_type |
| --- | --- | --- | --- | --- | --- | --- | --- | --- |
| Proboscis Length | 64 | 0.828 | 0.868 | ANOVA | 0.0003 | 0.0013 | 0.268 | η² |
| Forewing Length | 110 | 0.475 | 0.217 | ANOVA | 0 | 0.0001 | 0.221 | η² |
| Forewing Width | 110 | 0.411 | 0.384 | ANOVA | 0 | 0.0001 | 0.222 | η² |
| Vein Angle A4 | 110 | 0.002 | 0.011 | Kruskal-Wallis | 0.023 | 0.0507 | 0.062 | η²_H |
| Vein Angle B4 | 110 | 0.049 | 0.817 | Kruskal-Wallis | 0.3911 | 0.478 | 0 | η²_H |
| Vein Angle D7 | 110 | 0.311 | 0.86 | ANOVA | 0.0297 | 0.0613 | 0.081 | η² |
| Vein Angle E9 | 110 | 0.09 | 0.084 | ANOVA | 0 | 0.0001 | 0.235 | η² |
| Vein Angle J10 | 110 | 0.683 | 0.542 | ANOVA | 0.2572 | 0.3536 | 0.037 | η² |
| Vein Angle L13 | 110 | 0.358 | 0.887 | ANOVA | 0.8075 | 0.8075 | 0.009 | η² |
| Vein Angle J16 | 110 | 0.85 | 0.157 | ANOVA | 0.098 | 0.1469 | 0.057 | η² |
| Vein Angle G18 | 110 | 0.587 | 0.99 | ANOVA | 0.7576 | 0.7813 | 0.011 | η² |
| Vein Angle K19 | 110 | 0.274 | 0.555 | ANOVA | 0.7111 | 0.757 | 0.013 | η² |
| Vein Angle N23 | 110 | 0.84 | 0.085 | ANOVA | 0.0864 | 0.1358 | 0.06 | η² |
| Vein Angle O26 | 110 | 0.323 | 0.196 | ANOVA | 0.3797 | 0.478 | 0.028 | η² |
| Femur Length | 110 | 0.068 | 0.449 | ANOVA | 0.0005 | 0.002 | 0.152 | η² |
| Tibia Length | 110 | 0.485 | 0.156 | ANOVA | 0.0734 | 0.1211 | 0.063 | η² |
| Metatarsus Length | 110 | 0.637 | 0.247 | ANOVA | 0.0379 | 0.0703 | 0.076 | η² |
| Metatarsus Width | 110 | 0.434 | 0.429 | ANOVA | 0.1753 | 0.2515 | 0.045 | η² |
| Tergite 3 Longitudinal Length | 110 | 0.014 | 0.258 | Kruskal-Wallis | 0.0001 | 0.0007 | 0.171 | η²_H |
| Tergite 4 Longitudinal Length | 110 | 0.826 | 0.655 | ANOVA | 0.0003 | 0.0013 | 0.162 | η² |
| Tergite 5 Longitudinal Length | 110 | 0.003 | 0.503 | Kruskal-Wallis | 0.0139 | 0.0329 | 0.072 | η²_H |
| The Width of The Stripe of Tomentum on Tergite 5 | 110 | 0 | 0.56 | Kruskal-Wallis | 0.0439 | 0.0762 | 0.048 | η²_H |
| Tergite 6 Longitudinal Length | 110 | 0 | 0.031 | Kruskal-Wallis | 0.0001 | 0.0008 | 0.164 | η²_H |
| Length of Cover Hair on Tergite 6 | 110 | 0.087 | 0.991 | ANOVA | 0.0017 | 0.0047 | 0.132 | η² |
| Sternite 4 Longitudinal Length | 109 | 0.686 | 0.677 | ANOVA | 0.0012 | 0.0039 | 0.14 | η² |
| Length of Wax Plate on Sternite 4 | 109 | 0.745 | 0.458 | ANOVA | 0.0384 | 0.0703 | 0.077 | η² |
| Transverse Length of Wax Plate on Sternite 4 | 109 | 0.498 | 0.15 | ANOVA | 0.0014 | 0.0041 | 0.138 | η² |
| Distance between Wax Plate on Sternite 4 | 109 | 0.21 | 0.363 | ANOVA | 0.52 | 0.572 | 0.021 | η² |
| Sternite 7 Longitudinal Length | 108 | 0.066 | 0.635 | ANOVA | 0 | 0.0004 | 0.197 | η² |
| Sternite 7 Transverse Length | 109 | 0.963 | 0.476 | ANOVA | 0.0058 | 0.0148 | 0.112 | η² |
| Cubital Vein A | 109 | 0.12 | 0.573 | ANOVA | 0.345 | 0.4554 | 0.031 | η² |
| Cubital Vein B | 109 | 0.852 | 0.749 | ANOVA | 0.5135 | 0.572 | 0.022 | η² |
| Cubital Index | 109 | 0 | 0.708 | Kruskal-Wallis | 0.4139 | 0.4878 | 0 | η²_H |

**TABLE S2** | Genome-wide association study of morphological traits in honeybees from rocky desertification habitats in China.

| No. | Morphological traits | SNPs | QTN effects | LOD score | P value | Correlation |
| --- | --- | --- | --- | --- | --- | --- |
| 1 | proboscis length | Chr1_11280957 | -0.0399 | 8.0363 | 8.9292 | 10.0591 |
| 2 | proboscis length | Chr4_8719814 | 0.0306 | 8.5377 | 9.4431 | 5.3472 |
| 3 | proboscis length | Chr5_5375811 | 0.0372 | 6.4887 | 7.3377 | 7.6746 |
| 4 | proboscis length | Chr6_2658788 | 0.032 | 9.9617 | 10.8992 | 2.3093 |
| 5 | proboscis length | Chr8_22569417 | 0.0677 | 11.4835 | 12.4506 | 24.593 |
| 6 | proboscis length | Chr8_24219566 | -0.0369 | 8.081 | 8.9751 | 8.5927 |
| 7 | proboscis length | Chr8_25067043 | -0.0226 | 6.529 | 7.3793 | 3.375 |
| 8 | proboscis length | Chr9_3267132 | 0.0316 | 8.3431 | 9.2438 | 6.3307 |
| 9 | proboscis length | Chr9_6478388 | -0.0319 | 10.6964 | 11.6487 | 6.0974 |
| 10 | proboscis length | Chr16_10680287 | -0.0673 | 14.0532 | 15.0627 | 13.1552 |
| 11 | proboscis length | Chr2_12391460 | -0.0506 | 7.4002 | 8.2761 | 13.2699 |
| 12 | proboscis length | Chr2_7266779 | 0.0322 | 6.7884 | 7.6466 | 6.877 |
| 13 | proboscis length | Chr4_10315883 | -0.0571 | 7.3014 | 8.1746 | 13.2232 |
| 14 | proboscis length | Chr10_10482324 | -0.047 | 7.9958 | 8.8877 | 10.0262 |
| 15 | proboscis length | Chr15_1566150 | -0.0625 | 11.4017 | 12.3673 | 20.2629 |
| 16 | proboscis length | Chr16_3975576 | 0.0592 | 10.6068 | 11.5574 | 15.1033 |
| 17 | forewing length | Chr8_21469877 | -0.0588 | 7.0711 | 7.9377 | 7.3487 |
| 18 | forewing length | Chr16_11897263 | 0.1831 | 9.8199 | 10.7544 | 7.3852 |
| 19 | forewing length | Chr2_4283927 | -0.0523 | 6.4476 | 7.2953 | 3.1094 |
| 20 | forewing length | Chr8_13874564 | -0.047 | 6.715 | 7.571 | 4.4564 |
| 21 | forewing length | Chr6_13200793 | 0.0549 | 8.6002 | 9.5072 | 5.6283 |
| 22 | forewing length | Chr8_13875277 | -0.0293 | 7.6247 | 8.5068 | 1.8325 |
| 23 | forewing length | Chr8_26150562 | 0.0608 | 9.7626 | 10.6959 | 6.7057 |
| 24 | forewing length | Chr10_5111192 | 0.0504 | 11.8176 | 12.7907 | 6.8544 |
| 25 | forewing length | Chr15_5222542 | -0.0358 | 6.7113 | 7.5672 | 2.6636 |
| 26 | Forewing width | Chr8_24277727 | -0.0445 | 9.9729 | 10.9106 | 25.567 |
| 27 | Forewing width | Chr13_2585602 | 0.0214 | 7.2335 | 8.1048 | 7.9177 |
| 28 | Forewing width | Chr1_7142783 | -0.013 | 7.3643 | 8.2392 | 1.2221 |
| 29 | Forewing width | Chr8_24048516 | 0.0255 | 8.375 | 9.2765 | 4.4156 |
| 30 | Forewing width | Chr9_11448756 | -0.0144 | 9.3434 | 10.2675 | 3.6285 |
| 31 | Forewing width | Chr9_14773821 | -0.0184 | 6.4662 | 7.3145 | 5.8233 |
| 32 | Forewing width | Chr10_10934835 | 0.0179 | 12.6138 | 13.6006 | 5.0264 |
| 33 | Forewing width | Chr12_6431988 | 0.0123 | 10.8898 | 11.8458 | 1.6743 |
| 34 | Forewing width | Chr14_8912071 | -0.0138 | 6.6096 | 7.4624 | 1.9045 |
| 35 | Forewing width | Chr15_5041420 | 0.0209 | 9.5921 | 10.5217 | 6.1396 |
| 36 | Forewing width | Chr16_1342377 | 0.0104 | 8.67 | 9.5786 | 1.2752 |
| 37 | Forewing width | Chr16_12948819 | 0.0136 | 8.8901 | 9.8039 | 3.1833 |
| 38 | Forewing width | Chr3_702917 | 0.0301 | 8.0535 | 8.9469 | 8.6187 |
| 39 | Forewing width | Chr5_8755504 | 0.0178 | 6.6159 | 7.4689 | 4.9765 |
| 40 | Forewing width | Chr16_4712787 | 0.018 | 7.2382 | 8.1096 | 5.4707 |
| 41 | vein angle A4 | Chr3_3808879 | -0.7296 | 17.8919 | 18.9524 | 14.5438 |
| 42 | vein angle A4 | Chr5_2399156 | 0.3869 | 9.2005 | 10.1214 | 2.8516 |
| 43 | vein angle A4 | Chr6_4123438 | 0.3784 | 8.7222 | 9.6321 | 3.8067 |
| 44 | vein angle A4 | Chr7_10738916 | -0.4598 | 8.7558 | 9.6665 | 5.2682 |
| 45 | vein angle A4 | Chr8_8870502 | -0.555 | 17.403 | 18.4576 | 8.5956 |
| 46 | vein angle A4 | Chr8_25228265 | -0.5229 | 9.3224 | 10.2461 | 6.5188 |
| 47 | vein angle A4 | Chr8_5819554 | -0.4072 | 12.2101 | 13.1901 | 3.5947 |
| 48 | vein angle A4 | Chr10_5240069 | 0.6833 | 16.7564 | 17.803 | 12.4126 |
| 49 | vein angle A4 | Chr12_3928411 | 1.2335 | 17.3845 | 18.4389 | 16.5099 |
| 50 | vein angle A4 | Chr12_5688386 | 0.3233 | 9.9734 | 10.9111 | 1.9908 |
| 51 | vein angle A4 | Chr13_2585735 | -0.4289 | 11.5959 | 12.5651 | 3.9869 |
| 52 | vein angle A4 | Chr14_3301265 | -0.2901 | 7.0142 | 7.8791 | 2.3427 |
| 53 | vein angle A4 | Chr16_2609515 | -0.3692 | 10.4944 | 11.4427 | 3.7423 |
| 54 | vein angle A4 | Chr2_716406 | 0.6625 | 7.1693 | 8.0387 | 7.4676 |
| 55 | vein angle A4 | Chr4_243543 | 0.7552 | 9.023 | 9.9399 | 12.936 |
| 56 | vein angle A4 | Chr6_12953016 | -0.3387 | 6.4206 | 7.2674 | 2.17 |
| 57 | vein angle A4 | Chr8_14291272 | 0.5542 | 7.4124 | 8.2887 | 5.2249 |
| 58 | vein angle A4 | Chr10_9225702 | 0.4062 | 6.4165 | 7.2632 | 4.1539 |
| 59 | vein angle A4 | Chr14_6742486 | 0.7719 | 7.6996 | 8.5837 | 9.3267 |
| 60 | vein angle A4 | Chr15_2987915 | -0.856 | 7.4509 | 8.3282 | 8.7332 |
| 61 | vein angle A4 | Chr16_5165819 | 0.3802 | 6.9091 | 7.7709 | 2.553 |
| 62 | vein angle A4 | Chr16_6547327 | 1.3765 | 12.4429 | 13.4268 | 17.2221 |
| 63 | vein angle B4 | Chr1_2730496 | -0.8829 | 6.8436 | 7.7035 | 3.7351 |
| 64 | vein angle B4 | Chr1_15907230 | 1.1664 | 9.6446 | 10.5753 | 7.1861 |
| 65 | vein angle B4 | Chr1_7291771 | 0.9679 | 7.1149 | 7.9828 | 4.949 |
| 66 | vein angle B4 | Chr3_10308167 | 1.0744 | 8.5264 | 9.4316 | 3.7785 |
| 67 | vein angle B4 | Chr4_9857698 | -1.0204 | 8.2113 | 9.1087 | 5.5086 |
| 68 | vein angle B4 | Chr5_11947562 | 1.1497 | 7.8403 | 8.7281 | 5.1881 |
| 69 | vein angle B4 | Chr9_14689453 | 2.002 | 22.7171 | 23.8282 | 21.2065 |
| 70 | vein angle B4 | Chr9_5894110 | -0.9344 | 7.1735 | 8.0431 | 4.074 |
| 71 | vein angle B4 | Chr10_3474203 | 0.9377 | 8.0329 | 8.9258 | 3.5355 |
| 72 | vein angle B4 | Chr7_8927957 | 1.2615 | 6.462 | 7.3102 | 5.6046 |
| 73 | vein angle B4 | Chr13_2958118 | -2.7855 | 6.9752 | 7.839 | 15.6339 |
| 74 | vein angle B4 | Chr15_10761960 | 1.7426 | 10.7119 | 11.6645 | 13.4797 |
| 75 | vein angle B4 | Chr9_12880663 | -1.1267 | 7.2325 | 8.1037 | 6.0826 |
| 76 | vein angle B4 | Chr16_12980869 | -1.7404 | 10.3544 | 11.2999 | 9.9155 |
| 77 | vein angle B4 | Chr5_1939834 | -1.3783 | 6.466 | 7.3143 | 5.0043 |
| 78 | vein angle B4 | Chr9_10695550 | -1.733 | 10.2414 | 11.1846 | 11.7872 |
| 79 | vein angle B4 | Chr16_3926316 | 0.9856 | 9.7975 | 10.7315 | 2.6898 |
| 80 | vein angle B4 | Chr16_11738454 | -1.1521 | 9.5723 | 10.5015 | 5.8028 |
| 81 | vein angle D7 | Chr14_8574681 | 1.641 | 9.0988 | 10.0174 | 19.755 |
| 82 | vein angle D7 | Chr1_13754947 | 1.7636 | 9.0744 | 9.9925 | 16.3584 |
| 83 | vein angle D7 | Chr1_14626298 | 1.0562 | 8.3573 | 9.2583 | 8.2398 |
| 84 | vein angle D7 | Chr13_3870349 | 1.7793 | 7.2914 | 8.1643 | 14.754 |
| 85 | vein angle D7 | Chr9_2778953 | -2.417 | 7.9707 | 8.862 | 8.4384 |
| 86 | vein angle D7 | Chr5_3658660 | 0.9246 | 7.2362 | 8.1075 | 2.8585 |
| 87 | vein angle D7 | Chr13_2227545 | 1.5453 | 9.9646 | 10.9021 | 8.3251 |
| 88 | vein angle D7 | Chr1_14486940 | 0.8662 | 6.8556 | 7.7159 | 6.4978 |
| 89 | vein angle D7 | Chr6_2426563 | 2.1796 | 8.6266 | 9.5342 | 17.1495 |
| 90 | vein angle D7 | Chr6_12280302 | -1.3858 | 10.1296 | 11.0706 | 14.6462 |
| 91 | vein angle D7 | Chr7_3758251 | 1.3312 | 10.7021 | 11.6545 | 14.2051 |
| 92 | vein angle E9 | Chr1_770523 | -0.8475 | 10.8051 | 11.7595 | 9.0347 |
| 93 | vein angle E9 | Chr3_5153710 | 0.7856 | 10.9314 | 11.8882 | 7.2231 |
| 94 | vein angle E9 | Chr4_8176550 | 0.8942 | 9.2016 | 10.1226 | 4.2615 |
| 95 | vein angle E9 | Chr6_2018617 | 0.7609 | 10.4012 | 11.3477 | 6.5188 |
| 96 | vein angle E9 | Chr8_21719838 | -0.6438 | 6.8983 | 7.7598 | 3.6156 |
| 97 | vein angle E9 | Chr8_24046880 | 0.6634 | 7.2874 | 8.1602 | 5.4407 |
| 98 | vein angle E9 | Chr5_6708902 | 1.1453 | 9.6472 | 10.578 | 9.1802 |
| 99 | vein angle E9 | Chr6_2214301 | -0.8724 | 9.4058 | 10.3313 | 8.9062 |
| 100 | vein angle E9 | Chr10_10049509 | -0.9056 | 8.6849 | 9.5939 | 10.3143 |
| 101 | vein angle E9 | Chr15_7391168 | 0.795 | 6.6403 | 7.494 | 7.9089 |
| 102 | vein angle E9 | Chr13_1520358 | -1.5167 | 6.9164 | 7.7785 | 3.5267 |
| 103 | vein angle E9 | Chr16_4099687 | 0.9134 | 7.5618 | 8.4422 | 4.3729 |
| 104 | vein angle E9 | Chr4_1758276 | 0.9177 | 8.697 | 9.6063 | 7.9767 |
| 105 | vein angle E9 | Chr5_11457554 | 0.5853 | 9.5976 | 10.5273 | 3.9102 |
| 106 | vein angle E9 | Chr7_11571213 | 0.7758 | 7.1673 | 8.0367 | 6.2301 |
| 107 | vein angle E9 | Chr15_1142450 | 0.7225 | 6.9102 | 7.7721 | 2.9651 |
| 108 | vein angle J10 | Chr1_6421485 | 1.2761 | 9.2567 | 10.1789 | 10.9803 |
| 109 | vein angle J10 | Chr6_7003149 | 1.5513 | 6.6961 | 7.5515 | 9.2657 |
| 110 | vein angle J10 | Chr10_6006958 | 1.51 | 11.0354 | 11.9942 | 16.1245 |
| 111 | vein angle J10 | Chr12_4313969 | 1.3469 | 6.7964 | 7.6549 | 9.1182 |
| 112 | vein angle J10 | Chr15_8185003 | -1.2858 | 10.3084 | 11.253 | 9.4572 |
| 113 | vein angle J10 | Chr1_9220252 | 0.8157 | 7.1446 | 8.0133 | 3.3391 |
| 114 | vein angle J10 | Chr1_9832039 | -1.5796 | 10.1684 | 11.1102 | 15.8476 |
| 115 | vein angle J10 | Chr4_8312436 | 1.52 | 6.7436 | 7.6005 | 9.8569 |
| 116 | vein angle J10 | Chr10_4046117 | -0.8497 | 8.96 | 9.8755 | 5.384 |
| 117 | vein angle J10 | Chr10_10860281 | 1.0838 | 9.5355 | 10.4639 | 8.7581 |
| 118 | vein angle J10 | Chr15_9560394 | 1.0775 | 11.4817 | 12.4488 | 10.968 |
| 119 | vein angle L13 | Chr1_4713910 | -1.9676 | 8.9381 | 9.853 | 19.443 |
| 120 | vein angle L13 | Chr8_19740290 | -2.0599 | 8.2719 | 9.1708 | 5.8725 |
| 121 | vein angle L13 | Chr9_6783670 | -1.4985 | 6.9416 | 7.8044 | 12.4862 |
| 122 | vein angle L13 | Chr4_307907 | 2.2218 | 12.6008 | 13.5874 | 26.8768 |
| 123 | vein angle L13 | Chr9_3831992 | 1.3862 | 7.4036 | 8.2796 | 3.798 |
| 124 | vein angle L13 | Chr9_5638344 | 1.23 | 8.394 | 9.2959 | 8.4003 |
| 125 | vein angle L13 | Chr12_6245212 | -1.7855 | 9.612 | 10.542 | 15.6073 |
| 126 | vein angle L13 | Chr15_8774374 | 1.4088 | 9.8873 | 10.8232 | 11.0192 |
| 127 | vein angle J16 | Chr4_9229304 | -0.994 | 6.9142 | 7.7762 | 7.9398 |
| 128 | vein angle J16 | Chr9_12895608 | 1.2596 | 7.5936 | 8.4749 | 13.0772 |
| 129 | vein angle J16 | Chr13_10797318 | -1.0396 | 7.5295 | 8.409 | 6.4852 |
| 130 | vein angle J16 | Chr16_2936998 | 1.01 | 6.4981 | 7.3474 | 3.2961 |
| 131 | vein angle J16 | Chr2_10475967 | -1.1471 | 6.5288 | 7.3791 | 9.2652 |
| 132 | vein angle J16 | Chr3_10803619 | -0.996 | 9.6816 | 10.6131 | 5.1938 |
| 133 | vein angle J16 | Chr5_9541081 | 0.9527 | 7.1171 | 7.985 | 7.4234 |
| 134 | vein angle J16 | Chr14_1696932 | -1.0199 | 7.0253 | 7.8906 | 8.2814 |
| 135 | vein angle J16 | Chr16_6001060 | 1.144 | 8.8257 | 9.738 | 9.4284 |
| 136 | vein angle J16 | Chr16_13362593 | 0.9959 | 8.0972 | 8.9917 | 7.8457 |
| 137 | vein angle G18 | Chr1_880265 | 1.1647 | 6.7909 | 7.6492 | 9.5082 |
| 138 | vein angle G18 | Chr13_305886 | -1.4387 | 7.9134 | 8.8032 | 12.8156 |
| 139 | vein angle G18 | Chr15_8798549 | -1.2862 | 7.2125 | 8.0832 | 9.5971 |
| 140 | vein angle G18 | Chr2_11282528 | -0.7766 | 7.6299 | 8.5121 | 6.4544 |
| 141 | vein angle G18 | Chr3_2441404 | 1.3814 | 14.5534 | 15.5703 | 17.8704 |
| 142 | vein angle G18 | Chr6_14523163 | 1.0829 | 11.4408 | 12.4072 | 11.9473 |
| 143 | vein angle G18 | Chr7_1142323 | 0.7667 | 6.4812 | 7.33 | 6.0407 |
| 144 | vein angle G18 | Chr8_10390258 | 0.8692 | 10.4844 | 11.4325 | 8.1496 |
| 145 | vein angle G18 | Chr13_3907629 | -0.8664 | 9.5411 | 10.4696 | 5.8483 |
| 146 | vein angle G18 | Chr15_10158780 | 0.6726 | 7.686 | 8.5697 | 4.2995 |
| 147 | vein angle G18 | Chr13_8598511 | -2.4321 | 6.8974 | 7.7589 | 8.3298 |
| 148 | vein angle G18 | Chr2_4472747 | 0.7901 | 6.6184 | 7.4714 | 0.8959 |
| 149 | vein angle G18 | Chr1_15341392 | -1.1364 | 8.037 | 8.93 | 8.0054 |
| 150 | vein angle G18 | Chr12_3287071 | -0.9734 | 6.6858 | 7.5409 | 7.4312 |
| 151 | vein angle G18 | Chr14_2712297 | -0.7613 | 7.1498 | 8.0187 | 4.0536 |
| 152 | vein angle G18 | Chr15_10493 | 1.5317 | 8.2254 | 9.1231 | 12.4405 |
| 153 | vein angle K19 | Chr1_11868145 | -1.4198 | 12.4753 | 13.4598 | 15.9609 |
| 154 | vein angle K19 | Chr2_1914592 | -1.3234 | 17.2699 | 18.3229 | 18.2944 |
| 155 | vein angle K19 | Chr2_12215378 | -0.9926 | 6.9024 | 7.764 | 6.9154 |
| 156 | vein angle K19 | Chr7_2059676 | -0.7831 | 9.5859 | 10.5154 | 6.6452 |
| 157 | vein angle K19 | Chr8_23358654 | 0.6155 | 7.2001 | 8.0704 | 4.1493 |
| 158 | vein angle K19 | Chr9_2752061 | 0.6337 | 6.9308 | 7.7933 | 4.3905 |
| 159 | vein angle K19 | Chr11_5655094 | -0.6713 | 8.6593 | 9.5677 | 4.5751 |
| 160 | vein angle K19 | Chr12_2255874 | -1.0211 | 13.1702 | 14.1661 | 11.4321 |
| 161 | vein angle K19 | Chr12_4686409 | -1.1932 | 10.4546 | 11.4021 | 13.0544 |
| 162 | vein angle K19 | Chr14_4535362 | 1.5146 | 9.1 | 10.0187 | 15.4833 |
| 163 | vein angle K19 | Chr15_9886441 | -0.6373 | 8.3086 | 9.2084 | 3.9577 |
| 164 | vein angle K19 | Chr13_10951873 | -1.3339 | 7.0596 | 7.9259 | 5.8416 |
| 165 | vein angle K19 | Chr16_9888059 | -0.8815 | 8.1045 | 8.9992 | 7.8577 |
| 166 | vein angle K19 | Chr16_12363329 | 1.1177 | 6.574 | 7.4257 | 8.199 |
| 167 | vein angle N23 | Chr2_11113233 | 1.21 | 7.997 | 8.8889 | 22.0697 |
| 168 | vein angle N23 | Chr5_5889156 | -0.4079 | 7.2639 | 8.136 | 6.7331 |
| 169 | vein angle N23 | Chr9_9275099 | 0.5181 | 10.889 | 11.845 | 7.9971 |
| 170 | vein angle N23 | Chr11_4159438 | 0.4812 | 10.1635 | 11.1051 | 12.302 |
| 171 | vein angle N23 | Chr12_1830633 | -0.4188 | 8.8862 | 9.7999 | 10.0339 |
| 172 | vein angle N23 | Chr4_4771248 | 0.337 | 7.5679 | 8.4485 | 7.1856 |
| 173 | vein angle N23 | Chr8_5609550 | -0.4621 | 7.98 | 8.8715 | 10.3073 |
| 174 | vein angle N23 | Chr8_16295319 | -0.4168 | 7.2451 | 8.1167 | 9.4366 |
| 175 | vein angle N23 | Chr8_20949331 | -0.3529 | 7.9929 | 8.8847 | 7.8237 |
| 176 | vein angle N23 | Chr8_21113448 | 0.3375 | 6.9706 | 7.8343 | 1.9538 |
| 177 | vein angle N23 | Chr1_8722404 | 1.1329 | 9.8049 | 10.7391 | 5.5317 |
| 178 | vein angle N23 | Chr3_6459535 | 0.861 | 7.4548 | 8.3323 | 3.9936 |
| 179 | vein angle N23 | Chr14_3342320 | -0.8126 | 7.0722 | 7.9388 | 3.5087 |
| 180 | vein angle N23 | Chr5_10774268 | -0.4782 | 8.3009 | 9.2005 | 10.3842 |
| 181 | vein angle N23 | Chr9_10725326 | 0.709 | 8.6469 | 9.555 | 12.5509 |
| 182 | vein angle N23 | Chr16_13437420 | -0.4049 | 8.4119 | 9.3143 | 7.8385 |
| 183 | vein angle N23 | Chr1_12300214 | -0.3043 | 9.0612 | 9.979 | 5.3746 |
| 184 | vein angle N23 | Chr9_12133711 | -0.6803 | 10.591 | 11.5412 | 14.88 |
| 185 | vein angle N23 | Chr10_9452088 | 0.7255 | 11.1928 | 12.1546 | 15.3684 |
| 186 | vein angle N23 | Chr11_6075350 | -0.27 | 7.685 | 8.5687 | 2.2379 |
| 187 | vein angle O26 | Chr9_13338519 | -0.5942 | 7.457 | 8.3345 | 11.1338 |
| 188 | vein angle O26 | Chr12_1452233 | 0.4388 | 8.1298 | 9.0251 | 5.7527 |
| 189 | vein angle O26 | Chr16_6168024 | -0.6992 | 7.7685 | 8.6544 | 12.6768 |
| 190 | vein angle O26 | Chr16_12532862 | -0.361 | 6.6356 | 7.4892 | 3.0781 |
| 191 | vein angle O26 | Chr3_5039811 | -1.0817 | 6.9024 | 7.7641 | 6.77 |
| 192 | vein angle O26 | Chr15_6577036 | -1.2854 | 8.7814 | 9.6927 | 7.4449 |
| 193 | vein angle O26 | Chr1_12257365 | -0.6722 | 8.0311 | 8.9239 | 10.2944 |
| 194 | vein angle O26 | Chr9_13338486 | -0.6236 | 7.0328 | 7.8983 | 11.0215 |
| 195 | vein angle O26 | Chr15_6822392 | 0.5229 | 7.431 | 8.3078 | 8.166 |
| 196 | vein angle O26 | Chr16_10692660 | -0.6544 | 6.9984 | 7.8629 | 1.3644 |
| 197 | vein angle O26 | Chr1_12042383 | -0.4101 | 7.5227 | 8.402 | 7.0401 |
| 198 | vein angle O26 | Chr5_4330773 | 0.4717 | 6.5137 | 7.3635 | 5.8166 |
| 199 | vein angle O26 | Chr7_1096107 | -0.3802 | 8.866 | 9.7793 | 6.5753 |
| 200 | vein angle O26 | Chr7_6154287 | 0.3261 | 7.2301 | 8.1013 | 2.7794 |
| 201 | vein angle O26 | Chr7_7051169 | -0.9314 | 12.3402 | 13.3224 | 20.7334 |
| 202 | tibia length | Chr1_4141600 | 0.0218 | 6.8564 | 7.7167 | 7.2288 |
| 203 | tibia length | Chr1_14914611 | -0.0316 | 11.0649 | 12.0243 | 12.9174 |
| 204 | tibia length | Chr10_2599886 | -0.0207 | 9.4753 | 10.4024 | 6.9952 |
| 205 | tibia length | Chr13_3789198 | -0.0275 | 7.3796 | 8.255 | 8.4177 |
| 206 | tibia length | Chr14_6664193 | 0.0232 | 11.9922 | 12.9684 | 8.3594 |
| 207 | tibia length | Chr1_10984337 | 0.0412 | 7.6029 | 8.4844 | 10.7326 |
| 208 | tibia length | Chr8_4514381 | -0.0199 | 8.1141 | 9.009 | 2.3207 |
| 209 | tibia length | Chr11_7506734 | -0.0234 | 6.4514 | 7.2992 | 7.3627 |
| 210 | tibia length | Chr14_8751308 | 0.0264 | 8.9101 | 9.8244 | 10.6122 |
| 211 | tibia length | Chr1_13296701 | -0.0131 | 7.0196 | 7.8847 | 2.7849 |
| 212 | tibia length | Chr9_335471 | -0.0408 | 14.1739 | 15.1852 | 14.8844 |
| 213 | tibia length | Chr9_7394171 | -0.017 | 7.5945 | 8.4758 | 3.7956 |
| 214 | tibia length | Chr9_9575519 | 0.0289 | 16.4741 | 17.5171 | 1.9109 |
| 215 | tibia length | Chr9_11444611 | 0.0156 | 7.1694 | 8.0388 | 2.8572 |
| 216 | tibia length | Chr14_9112897 | 0.0227 | 17.0222 | 18.0721 | 7.9004 |
| 217 | femur length | Chr8_16934859 | 0.0684 | 12.879 | 13.8702 | 25.975 |
| 218 | femur length | Chr5_6248904 | 0.0568 | 7.1509 | 8.0198 | 3.9463 |
| 219 | femur length | Chr6_8785489 | -0.0337 | 7.3572 | 8.2319 | 2.746 |
| 220 | femur length | Chr2_651232 | 0.0228 | 8.4564 | 9.3599 | 5.5376 |
| 221 | femur length | Chr5_10054755 | -0.0217 | 10.6064 | 11.5569 | 5.0202 |
| 222 | femur length | Chr7_6282822 | -0.0244 | 8.817 | 9.7291 | 5.6791 |
| 223 | femur length | Chr8_9350419 | 0.0379 | 19.9141 | 20.9972 | 14.4735 |
| 224 | femur length | Chr9_2238312 | 0.0257 | 8.3713 | 9.2727 | 5.3661 |
| 225 | femur length | Chr11_7656129 | -0.0344 | 8.4905 | 9.3948 | 6.9275 |
| 226 | femur length | Chr13_4676097 | -0.0246 | 10.7398 | 11.693 | 6.3255 |
| 227 | femur length | Chr13_6664106 | -0.0302 | 8.1269 | 9.0222 | 6.8333 |
| 228 | femur length | Chr13_8717059 | 0.015 | 8.1987 | 9.0958 | 1.9723 |
| 229 | femur length | Chr16_149412 | 0.0306 | 7.078 | 7.9448 | 5.2448 |
| 230 | femur length | Chr16_6263559 | 0.0381 | 16.4533 | 17.496 | 11.4811 |
| 231 | metatarsus length | Chr7_2913902 | 0.0203 | 9.9332 | 10.8701 | 7.1672 |
| 232 | metatarsus length | Chr7_4488377 | 0.0169 | 8.4048 | 9.307 | 5.9254 |
| 233 | metatarsus length | Chr9_10212824 | 0.0238 | 8.564 | 9.4701 | 11.7986 |
| 234 | metatarsus length | Chr9_13913099 | 0.0187 | 9.0065 | 9.923 | 6.6697 |
| 235 | metatarsus length | Chr12_6862264 | -0.0218 | 8.9563 | 9.8717 | 7.7628 |
| 236 | metatarsus length | Chr13_2682606 | -0.0233 | 6.5889 | 7.441 | 6.5231 |
| 237 | metatarsus length | Chr4_4923237 | 0.014 | 7.2551 | 8.127 | 2.0673 |
| 238 | metatarsus length | Chr10_7148567 | -0.0266 | 9.755 | 10.6881 | 9.4345 |
| 239 | metatarsus length | Chr15_5610401 | -0.0161 | 7.0701 | 7.9367 | 3.8226 |
| 240 | metatarsus length | Chr16_6554808 | -0.0115 | 6.7785 | 7.6364 | 1.5274 |
| 241 | metatarsus length | Chr6_5458221 | -0.0402 | 12.6316 | 13.6187 | 16.3569 |
| 242 | metatarsus length | Chr9_13543880 | 0.026 | 7.1428 | 8.0115 | 7.8492 |
| 243 | metatarsus length | Chr11_3613559 | -0.0177 | 6.4938 | 7.343 | 6.0112 |
| 244 | metatarsus width | Chr3_9964245 | -0.0153 | 6.8679 | 7.7285 | 3.9221 |
| 245 | metatarsus width | Chr5_7704262 | 0.0136 | 10.2635 | 11.2072 | 6.9698 |
| 246 | metatarsus width | Chr8_3122336 | 0.0128 | 18.5927 | 19.6613 | 5.3416 |
| 247 | metatarsus width | Chr8_12422129 | 0.008 | 9.0103 | 9.9269 | 2.2657 |
| 248 | metatarsus width | Chr8_20859973 | -0.006 | 8.4195 | 9.3221 | 1.2939 |
| 249 | metatarsus width | Chr8_25965418 | 0.0152 | 9.204 | 10.125 | 8.232 |
| 250 | metatarsus width | Chr13_4817853 | 0.0133 | 6.8323 | 7.6919 | 5.4362 |
| 251 | metatarsus width | Chr15_9248668 | 0.012 | 7.3523 | 8.2269 | 4.3893 |
| 252 | metatarsus width | Chr6_14027019 | -0.0114 | 7.2225 | 8.0934 | 4.7933 |
| 253 | metatarsus width | Chr7_11927570 | -0.0116 | 7.2825 | 8.1551 | 1.2018 |
| 254 | metatarsus width | Chr16_4590070 | -0.0173 | 9.24 | 10.1618 | 10.6199 |
| 255 | metatarsus width | Chr3_1911575 | 0.0121 | 6.9976 | 7.8621 | 5.1602 |
| 256 | metatarsus width | Chr5_2127058 | -0.0106 | 7.2146 | 8.0853 | 3.3472 |
| 257 | metatarsus width | Chr6_6437543 | 0.0141 | 6.9975 | 7.862 | 6.9131 |
| 258 | metatarsus width | Chr8_17370061 | 0.0066 | 7.8168 | 8.704 | 1.6291 |
| 259 | metatarsus width | Chr10_6873045 | -0.0179 | 10.2847 | 11.2288 | 10.4944 |
| 260 | metatarsus width | Chr10_11597136 | -0.0123 | 7.334 | 8.2081 | 5.4235 |
| 261 | metatarsus width | Chr13_476173 | -0.0133 | 10.7295 | 11.6825 | 3.999 |
| 262 | metatarsus width | Chr13_1711920 | -0.0278 | 11.4023 | 12.368 | 12.1391 |
| 263 | metatarsus width | Chr13_10019403 | 0.0163 | 8.431 | 9.3338 | 5.7682 |
| 264 | longitudinal lengths of tergite 3 | Chr1_8475007 | 0.0266 | 8.0775 | 8.9715 | 6.8807 |
| 265 | longitudinal lengths of tergite 3 | Chr2_12154973 | 0.0379 | 7.7492 | 8.6346 | 9.616 |
| 266 | longitudinal lengths of tergite 3 | Chr8_15093812 | 0.0324 | 9.9391 | 10.8761 | 9.0196 |
| 267 | longitudinal lengths of tergite 3 | Chr8_20196095 | -0.0392 | 8.0663 | 8.96 | 8.6111 |
| 268 | longitudinal lengths of tergite 3 | Chr11_3998724 | 0.0345 | 7.1317 | 8.0001 | 9.5378 |
| 269 | longitudinal lengths of tergite 3 | Chr14_2489590 | 0.0262 | 7.2423 | 8.1138 | 8.7841 |
| 270 | longitudinal lengths of tergite 3 | Chr6_12211745 | -0.0297 | 10.7504 | 11.7038 | 0.5212 |
| 271 | longitudinal lengths of tergite 3 | Chr3_4312043 | -0.0253 | 7.3187 | 8.1924 | 5.1873 |
| 272 | longitudinal lengths of tergite 3 | Chr6_2202840 | 0.0325 | 9.6806 | 10.6121 | 8.5549 |
| 273 | longitudinal lengths of tergite 3 | Chr7_2399313 | 0.0443 | 14.4962 | 15.5122 | 12.5891 |
| 274 | longitudinal lengths of tergite 3 | Chr8_3887146 | -0.0241 | 9.3052 | 10.2285 | 7.6884 |
| 275 | longitudinal lengths of tergite 3 | Chr8_4153754 | 0.029 | 7.5118 | 8.3908 | 6.2986 |
| 276 | longitudinal lengths of tergite 3 | Chr10_4953041 | 0.0239 | 10.1545 | 11.096 | 7.6548 |
| 277 | longitudinal lengths of tergite 3 | Chr11_8060934 | 0.021 | 11.3663 | 12.3313 | 6.0029 |
| 278 | longitudinal lengths of tergite 4 | Chr1_10726782 | 0.0124 | 8.5941 | 9.5009 | 3.098 |
| 279 | longitudinal lengths of tergite 4 | Chr2_4606703 | -0.0154 | 14.4638 | 15.4794 | 7.6627 |
| 280 | longitudinal lengths of tergite 4 | Chr5_5526594 | -0.0051 | 7.9474 | 8.838 | 0.9875 |
| 281 | longitudinal lengths of tergite 4 | Chr8_25373310 | 0.0064 | 7.4927 | 8.3712 | 1.6779 |
| 282 | longitudinal lengths of tergite 4 | Chr8_25571409 | 0.0165 | 10.8277 | 11.7826 | 9.0664 |
| 283 | longitudinal lengths of tergite 4 | Chr9_6012775 | 0.022 | 14.6724 | 15.691 | 10.2194 |
| 284 | longitudinal lengths of tergite 4 | Chr10_2132138 | -0.01 | 16.6129 | 17.6577 | 4.0297 |
| 285 | longitudinal lengths of tergite 4 | Chr15_9438186 | 0.0115 | 10.7091 | 11.6617 | 5.3852 |
| 286 | longitudinal lengths of tergite 4 | Chr16_2957280 | 0.0099 | 11.6234 | 12.5931 | 3.91 |
| 287 | longitudinal lengths of tergite 4 | Chr3_1832013 | -0.0129 | 7.0154 | 7.8804 | 5.2406 |
| 288 | longitudinal lengths of tergite 4 | Chr3_9964245 | -0.0181 | 10.1809 | 11.1229 | 5.9123 |
| 289 | longitudinal lengths of tergite 4 | Chr8_15372087 | -0.0091 | 7.7548 | 8.6404 | 3.2715 |
| 290 | longitudinal lengths of tergite 4 | Chr9_12057072 | -0.0104 | 6.6695 | 7.5241 | 4.3689 |
| 291 | longitudinal lengths of tergite 4 | Chr13_6796402 | -0.0116 | 11.746 | 12.7179 | 3.4644 |
| 292 | longitudinal lengths of tergite 4 | Chr13_12037802 | -0.0313 | 7.9656 | 8.8567 | 10.6852 |
| 293 | longitudinal lengths of tergite 4 | Chr3_3465613 | -0.0115 | 7.3057 | 8.179 | 5.3091 |
| 294 | longitudinal lengths of tergite 4 | Chr12_6847319 | -0.0201 | 8.849 | 9.7619 | 10.8674 |
| 295 | longitudinal lengths of tergite 4 | Chr15_4553051 | -0.0127 | 9.618 | 10.5482 | 6.3677 |
| 296 | longitudinal lengths of tergite 5 | Chr9_2793976 | -0.0176 | 8.9359 | 9.8508 | 7.6035 |
| 297 | longitudinal lengths of tergite 5 | Chr10_3724460 | 0.0288 | 6.8891 | 7.7504 | 10.6463 |
| 298 | longitudinal lengths of tergite 5 | Chr15_5236532 | 0.026 | 6.7316 | 7.5881 | 13.3033 |
| 299 | longitudinal lengths of tergite 5 | Chr2_5367052 | 0.0128 | 7.4017 | 8.2777 | 2.5106 |
| 300 | longitudinal lengths of tergite 5 | Chr6_14055927 | -0.0228 | 8.3602 | 9.2613 | 6.1462 |
| 301 | longitudinal lengths of tergite 5 | Chr16_9961744 | 0.0229 | 7.8932 | 8.7824 | 4.5741 |
| 302 | longitudinal lengths of tergite 5 | Chr14_9113935 | 0.013 | 6.585 | 7.437 | 4.6695 |
| 303 | width of the tomentum stripe on tergite 5 | Chr5_3933005 | 0.0376 | 8.1261 | 9.0213 | 7.022 |
| 304 | width of the tomentum stripe on tergite 5 | Chr5_5421486 | -0.04 | 7.5183 | 8.3975 | 7.621 |
| 305 | width of the tomentum stripe on tergite 5 | Chr8_9119684 | -0.0455 | 8.3182 | 9.2183 | 8.3257 |
| 306 | width of the tomentum stripe on tergite 5 | Chr8_23369365 | 0.0559 | 7.0184 | 7.8835 | 8.103 |
| 307 | width of the tomentum stripe on tergite 5 | Chr12_2784319 | -0.0538 | 7.6019 | 8.4834 | 8.8713 |
| 308 | width of the tomentum stripe on tergite 5 | Chr2_372448 | 0.053 | 18.9211 | 19.9934 | 17.6175 |
| 309 | width of the tomentum stripe on tergite 5 | Chr2_3182340 | 0.0366 | 7.3434 | 8.2178 | 7.6848 |
| 310 | width of the tomentum stripe on tergite 5 | Chr2_10751395 | 0.0443 | 8.8723 | 9.7857 | 8.4057 |
| 311 | width of the tomentum stripe on tergite 5 | Chr4_250733 | -0.0514 | 9.7917 | 10.7256 | 4.9943 |
| 312 | width of the tomentum stripe on tergite 5 | Chr7_2641721 | -0.0289 | 7.0669 | 7.9334 | 4.6161 |
| 313 | width of the tomentum stripe on tergite 5 | Chr13_2125438 | 0.0483 | 9.2679 | 10.1904 | 9.3798 |
| 314 | width of the tomentum stripe on tergite 5 | Chr16_1252077 | 0.0493 | 7.6735 | 8.5569 | 7.9678 |
| 315 | width of the tomentum stripe on tergite 5 | Chr16_11692520 | 0.0386 | 9.5485 | 10.4772 | 6.3879 |
| 316 | longitudinal lengths of tergite 6 | Chr1_12663716 | 0.0725 | 15.746 | 16.7795 | 20.7154 |
| 317 | longitudinal lengths of tergite 6 | Chr2_8384491 | -0.0461 | 16.0931 | 17.1312 | 4.7837 |
| 318 | longitudinal lengths of tergite 6 | Chr3_6186416 | -0.032 | 8.7926 | 9.7041 | 5.989 |
| 319 | longitudinal lengths of tergite 6 | Chr5_6270881 | 0.0361 | 7.1867 | 8.0566 | 5.4837 |
| 320 | longitudinal lengths of tergite 6 | Chr7_1540780 | 0.0179 | 6.4473 | 7.295 | 2.9499 |
| 321 | longitudinal lengths of tergite 6 | Chr8_23148541 | 0.0537 | 11.115 | 12.0753 | 14.281 |
| 322 | longitudinal lengths of tergite 6 | Chr11_5709073 | 0.0233 | 6.7253 | 7.5816 | 4.0767 |
| 323 | longitudinal lengths of tergite 6 | Chr13_7686105 | 0.0443 | 9.8615 | 10.7969 | 10.6299 |
| 324 | longitudinal lengths of tergite 6 | Chr14_8338057 | 0.0503 | 8.4138 | 9.3162 | 9.3054 |
| 325 | longitudinal lengths of tergite 6 | Chr6_5682594 | -0.1068 | 8.5739 | 9.4802 | 8.951 |
| 326 | longitudinal lengths of tergite 6 | Chr13_5417875 | 0.0671 | 8.26 | 9.1586 | 4.9767 |
| 327 | longitudinal lengths of tergite 6 | Chr2_3072141 | 0.0338 | 12.4286 | 13.4123 | 7.8548 |
| 328 | longitudinal lengths of tergite 6 | Chr3_4941561 | -0.023 | 9.5732 | 10.5024 | 5.0482 |
| 329 | longitudinal lengths of tergite 6 | Chr4_8652234 | 0.034 | 15.2523 | 16.2791 | 9.2618 |
| 330 | longitudinal lengths of tergite 6 | Chr7_5649194 | -0.0155 | 6.5888 | 7.4409 | 0.8908 |
| 331 | longitudinal lengths of tergite 6 | Chr8_8798771 | -0.0317 | 9.9617 | 10.8992 | 7.7831 |
| 332 | longitudinal lengths of tergite 6 | Chr13_8219696 | 0.0332 | 9.9419 | 10.879 | 9.5312 |
| 333 | length of cover hairs on tergite 6 | Chr6_6077179 | -0.0338 | 8.9811 | 9.897 | 11.4174 |
| 334 | length of cover hairs on tergite 6 | Chr7_1345773 | 0.0315 | 9.127 | 10.0463 | 4.7986 |
| 335 | length of cover hairs on tergite 6 | Chr9_13683140 | -0.0534 | 9.0771 | 9.9952 | 17.7654 |
| 336 | length of cover hairs on tergite 6 | Chr12_2634041 | -0.0336 | 12.2993 | 13.2808 | 12.3254 |
| 337 | length of cover hairs on tergite 6 | Chr13_1343523 | -0.0331 | 7.1113 | 7.9791 | 7.6332 |
| 338 | length of cover hairs on tergite 6 | Chr7_5091951 | -0.0241 | 6.6549 | 7.5091 | 5.6771 |
| 339 | length of cover hairs on tergite 6 | Chr3_2063601 | -0.0415 | 8.7226 | 9.6325 | 9.0639 |
| 340 | length of cover hairs on tergite 6 | Chr4_11164976 | -0.022 | 6.4216 | 7.2685 | 5.7502 |
| 341 | length of cover hairs on tergite 6 | Chr7_3696742 | -0.0449 | 7.2649 | 8.1371 | 9.9629 |
| 342 | length of cover hairs on tergite 6 | Chr7_10111081 | -0.0189 | 7.2613 | 8.1333 | 1.7705 |
| 343 | length of cover hairs on tergite 6 | Chr11_808828 | -0.0403 | 7.4191 | 8.2956 | 9.09 |
| 344 | length of cover hairs on tergite 6 | Chr16_11732362 | 0.0258 | 7.6167 | 8.4986 | 7.537 |
| 345 | longitudinal length of sternite 4 | Chr1_471180 | -0.0181 | 7.1723 | 8.0418 | 6.2877 |
| 346 | longitudinal length of sternite 4 | Chr12_1162897 | 0.0287 | 7.0241 | 7.8893 | 8.0003 |
| 347 | longitudinal length of sternite 4 | Chr13_6541768 | 0.02 | 6.9106 | 7.7725 | 6.8295 |
| 348 | longitudinal length of sternite 4 | Chr14_6191002 | -0.0156 | 6.8915 | 7.7528 | 1.2845 |
| 349 | longitudinal length of sternite 4 | Chr8_1392239 | -0.0307 | 8.2462 | 9.1445 | 12.1632 |
| 350 | longitudinal length of sternite 4 | Chr16_1590796 | 0.0212 | 7.3652 | 8.2402 | 0.931 |
| 351 | length of wax plate on sternite 4 | Chr3_4704155 | -0.0131 | 9.6976 | 10.6295 | 4.7283 |
| 352 | length of wax plate on sternite 4 | Chr5_8938327 | -0.0214 | 16.5853 | 17.6297 | 10.4146 |
| 353 | length of wax plate on sternite 4 | Chr5_12562373 | 0.0205 | 6.9255 | 7.7878 | 6.6257 |
| 354 | length of wax plate on sternite 4 | Chr6_2431887 | -0.0278 | 16.9824 | 18.0318 | 16.583 |
| 355 | length of wax plate on sternite 4 | Chr8_11733456 | 0.0126 | 9.3322 | 10.2561 | 3.8079 |
| 356 | length of wax plate on sternite 4 | Chr10_4724541 | 0.0121 | 7.5944 | 8.4757 | 4.0387 |
| 357 | length of wax plate on sternite 4 | Chr10_7038856 | 0.0121 | 11.3363 | 12.3007 | 4.7093 |
| 358 | length of wax plate on sternite 4 | Chr13_2862350 | 0.015 | 6.5664 | 7.4178 | 6.572 |
| 359 | length of wax plate on sternite 4 | Chr2_12096445 | -0.0052 | 6.6225 | 7.4757 | 0.8069 |
| 360 | length of wax plate on sternite 4 | Chr7_6111867 | -0.0102 | 6.7038 | 7.5595 | 2.0158 |
| 361 | length of wax plate on sternite 4 | Chr11_935310 | -0.011 | 10.5991 | 11.5495 | 2.7424 |
| 362 | length of wax plate on sternite 4 | Chr14_831871 | 0.0095 | 7.1998 | 8.0701 | 2.657 |
| 363 | length of wax plate on sternite 4 | Chr16_6452734 | 0.0158 | 7.0278 | 7.8931 | 5.1587 |
| 364 | length of wax plate on sternite 4 | Chr5_4809761 | 0.018 | 6.8743 | 7.7351 | 8.9518 |
| 365 | transverse length of wax plate on sternite 4 | Chr8_25747280 | -0.0274 | 6.7136 | 7.5696 | 7.4057 |
| 366 | transverse length of wax plate on sternite 4 | Chr13_3258617 | -0.0283 | 6.85 | 7.7101 | 11.4275 |
| 367 | transverse length of wax plate on sternite 4 | Chr3_441324 | 0.0241 | 9.5178 | 10.4458 | 6.1415 |
| 368 | transverse length of wax plate on sternite 4 | Chr7_12087212 | 0.0131 | 8.5011 | 9.4057 | 2.3609 |
| 369 | transverse length of wax plate on sternite 4 | Chr16_5578289 | -0.023 | 8.7326 | 9.6427 | 10.954 |
| 370 | transverse length of wax plate on sternite 4 | Chr16_9369077 | -0.0165 | 9.1383 | 10.0578 | 5.6444 |
| 371 | transverse length of wax plate on sternite 4 | Chr16_10187220 | -0.0184 | 7.4501 | 8.3274 | 7.1183 |
| 372 | transverse length of wax plate on sternite 4 | Chr1_8107607 | 0.0311 | 8.633 | 9.5407 | 12.1765 |
| 373 | transverse length of wax plate on sternite 4 | Chr4_9798167 | 0.0281 | 7.5208 | 8.4001 | 11.6377 |
| 374 | transverse length of wax plate on sternite 4 | Chr6_13146347 | -0.0284 | 6.6564 | 7.5106 | 8.5924 |
| 375 | transverse length of wax plate on sternite 4 | Chr12_5408316 | 0.0343 | 6.6533 | 7.5074 | 11.9393 |
| 376 | transverse length of wax plate on sternite 4 | Chr15_8741554 | -0.0211 | 9.695 | 10.6268 | 9.0052 |
| 377 | distance between wax plates on sternite 4 | Chr11_8009705 | -0.0292 | 8.0181 | 8.9106 | 12.7783 |
| 378 | distance between wax plates on sternite 4 | Chr1_3672122 | -0.0183 | 10.4486 | 11.396 | 5.3746 |
| 379 | distance between wax plates on sternite 4 | Chr1_14823203 | 0.0127 | 6.4671 | 7.3154 | 2.5183 |
| 380 | distance between wax plates on sternite 4 | Chr2_5858333 | -0.0179 | 11.3975 | 12.3631 | 5.2924 |
| 381 | distance between wax plates on sternite 4 | Chr7_11291599 | -0.0212 | 9.1274 | 10.0467 | 4.9248 |
| 382 | distance between wax plates on sternite 4 | Chr8_7107313 | 0.0266 | 8.0485 | 8.9418 | 4.3809 |
| 383 | distance between wax plates on sternite 4 | Chr9_3192512 | -0.0137 | 10.538 | 11.4872 | 1.9182 |
| 384 | distance between wax plates on sternite 4 | Chr11_6843580 | -0.016 | 6.9557 | 7.8189 | 2.694 |
| 385 | distance between wax plates on sternite 4 | Chr12_2482980 | -0.0116 | 7.1794 | 8.0491 | 2.2632 |
| 386 | distance between wax plates on sternite 4 | Chr15_7024667 | -0.0115 | 6.926 | 7.7883 | 2.1463 |
| 387 | distance between wax plates on sternite 4 | Chr4_11220233 | -0.0123 | 7.0189 | 7.884 | 3.8678 |
| 388 | distance between wax plates on sternite 4 | Chr6_7034795 | 0.0168 | 6.7351 | 7.5917 | 7.4008 |
| 389 | distance between wax plates on sternite 4 | Chr6_11831246 | 0.0115 | 7.4851 | 8.3634 | 4.7703 |
| 390 | distance between wax plates on sternite 4 | Chr6_13416426 | 0.0101 | 6.9693 | 7.8329 | 2.8197 |
| 391 | distance between wax plates on sternite 4 | Chr8_10586072 | -0.0134 | 6.7812 | 7.6392 | 6.4784 |
| 392 | distance between wax plates on sternite 4 | Chr9_704580 | 0.0148 | 7.0677 | 7.9342 | 3.9452 |
| 393 | distance between wax plates on sternite 4 | Chr11_7784524 | -0.0198 | 8.2787 | 9.1778 | 11.1244 |
| 394 | longitudinal length of sternite 7 | Chr4_1310479 | -0.0307 | 7.6437 | 8.5263 | 8.9889 |
| 395 | longitudinal length of sternite 7 | Chr8_1913157 | -0.0327 | 7.2362 | 8.1075 | 9.2413 |
| 396 | longitudinal length of sternite 7 | Chr9_10812095 | -0.0152 | 7.3723 | 8.2475 | 3.6463 |
| 397 | longitudinal length of sternite 7 | Chr13_4834814 | 0.0251 | 12.8705 | 13.8615 | 11.1351 |
| 398 | longitudinal length of sternite 7 | Chr1_3498307 | -0.0309 | 9.2387 | 10.1605 | 6.5096 |
| 399 | longitudinal length of sternite 7 | Chr3_1449761 | -0.0181 | 6.9674 | 7.831 | 3.2075 |
| 400 | longitudinal length of sternite 7 | Chr4_8403242 | 0.0287 | 7.4264 | 8.3031 | 4.3214 |
| 401 | longitudinal length of sternite 7 | Chr6_6922463 | -0.0184 | 7.1097 | 7.9774 | 3.1347 |
| 402 | longitudinal length of sternite 7 | Chr16_10292094 | -0.0206 | 7.9479 | 8.8386 | 4.1811 |
| 403 | longitudinal length of sternite 7 | Chr6_7564912 | -0.023 | 7.4858 | 8.3641 | 5.281 |
| 404 | longitudinal length of sternite 7 | Chr8_2973756 | 0.0174 | 7.7967 | 8.6834 | 0.9615 |
| 405 | longitudinal length of sternite 7 | Chr8_22605012 | 0.0245 | 11.7718 | 12.7441 | 10.2663 |
| 406 | longitudinal length of sternite 7 | Chr14_1713855 | 0.021 | 8.6731 | 9.5818 | 6.6427 |
| 407 | longitudinal length of sternite 7 | Chr16_2688825 | 0.0164 | 7.9446 | 8.8352 | 4.8589 |
| 408 | transverse length of sternite 7 | Chr1_15058646 | -0.0194 | 7.4924 | 8.3709 | 0.6204 |
| 409 | transverse length of sternite 7 | Chr2_4240333 | -0.0224 | 11.2766 | 12.2399 | 4.8029 |
| 410 | transverse length of sternite 7 | Chr6_14035973 | -0.0164 | 8.715 | 9.6247 | 3.995 |
| 411 | transverse length of sternite 7 | Chr8_438895 | 0.0232 | 6.5194 | 7.3694 | 3.921 |
| 412 | transverse length of sternite 7 | Chr8_15232667 | -0.033 | 15.7003 | 16.7332 | 14.5589 |
| 413 | transverse length of sternite 7 | Chr14_1190990 | -0.0157 | 7.881 | 8.7699 | 3.7931 |
| 414 | transverse length of sternite 7 | Chr14_8470190 | -0.0296 | 8.7371 | 9.6473 | 8.3503 |
| 415 | transverse length of sternite 7 | Chr16_5420202 | 0.0172 | 7.3325 | 8.2066 | 4.4775 |
| 416 | transverse length of sternite 7 | Chr4_7452074 | -0.0179 | 6.9271 | 7.7895 | 1.0565 |
| 417 | transverse length of sternite 7 | Chr5_5133909 | -0.0295 | 6.7507 | 7.6078 | 6.3328 |
| 418 | transverse length of sternite 7 | Chr8_6346894 | -0.0187 | 8.689 | 9.5981 | 3.6533 |
| 419 | transverse length of sternite 7 | Chr15_1160311 | -0.0279 | 9.127 | 10.0463 | 7.0635 |
| 420 | transverse length of sternite 7 | Chr4_1710055 | 0.0281 | 9.9235 | 10.8602 | 12.1137 |
| 421 | transverse length of sternite 7 | Chr10_7820660 | -0.0257 | 6.4797 | 7.3284 | 9.0977 |

**FIGURE S1 |** Linear mixed model (LMM) estimation of proportional variance among-groups and within-groups based on honeybee morphology in rocky desertification habitats.
